# Epigenetic subtypes of high-grade T1 bladder cancer reveal intra-tumor heterogeneity and distinct interactions with tumor microenvironment

**DOI:** 10.7150/thno.129729

**Published:** 2026-05-18

**Authors:** Joaquim Bellmunt, Yingtian Xie, Nuria Juanpere, Miguel Gomez Munoz, Shweta Kukreja, Sonsoles Liria Veiga, Rong Li, Xintao Qiu, Yijia Jiang, Alba Font-Tello, Marie Nunez Duarte, Ilana Epstein, Silvia Hernández-Llodrà, Marta Lorenzo, Silvia Menendez, Toni Choueiri, Myles Brown, Henry W Long, Paloma Cejas

**Affiliations:** 1 Harvard Medical School, Boston, MA, USA; Lank Center for Genitourinary Oncology, Dana-Farber Cancer Institute, Boston, MA, USA.; 2 Center for Functional Cancer Epigenetics, Dana-Farber Cancer Institute, Boston, MA, 02215, USA.; 3 Department of Pathology, Hospital del Mar, Barcelona, Spain.; 4 Translational Oncology Laboratory, Hospital La Paz Institute for Health Research, (IdiPAZ), Madrid, Spain.; 5 Department of Medicine and Life Sciences, Universitat Pompeu Fabra, Barcelona, Spain.

## Abstract

**Rationale:**

High-grade T1 (HGT1) Non-Muscle Invasive Bladder Cancer (NMIBC) is a clinically heterogeneous disease characterized by unpredictable treatment responses and limited tools for recurrence prediction. Despite advances in molecular classification, patient stratification still relies mainly on clinicopathological features, providing limited precision.

**Methods:**

In this study, we integrated bulk chromatin profiling with single-nuclei RNA-seq, immunohistochemistry, and spatial transcriptomics to define epigenetic subtypes of HGT1, characterize their heterogeneity, and explore tumor–microenvironment interactions.

**Results:**

We identified distinct chromatin landscapes distinguishing urothelial (URO) and micropapillary (MP) histological variants of high-risk HGT1. Three main epigenetic states emerged: luminal-like inflammatory (LLI) and basal-like (BL) subtypes within URO tumors, and a separate signature unique to MP tumors. Single-cell and spatial analyses confirmed intratumoral heterogeneity and revealed subtype-specific microenvironmental contexts.

Approximately 40% of URO tumors exhibited spatially distinct coexisting LLI and BL components. BL regions, enriched for angiogenesis and hypoxia pathways, were preferentially located near vascular stroma, whereas LLI regions were situated at the tumor core. MP tumors displayed a markedly different microenvironment with abundant cancer-associated fibroblasts (CAFs) and M2-polarized macrophages intermingled with tumor cells, suggesting an immunosuppressive niche that may underlie their poor prognosis. In contrast, URO tumors showed a more immune-excluded phenotype.

**Conclusion:**

These findings provide a detailed molecular and spatial map of HGT1 heterogeneity, linking epigenetic states to tumor architecture and microenvironmental interactions. They underscore the need for subtype-specific therapeutic strategies to effectively address the biological diversity already present in HGT1 bladder cancer.

## Introduction

Non-muscle invasive bladder cancer (NMIBC) is an early-stage bladder cancer that shows heterogeneity in terms of disease outcomes. High-grade T1 (HGT1) NMIBC has the highest risk of disease recurrence (approximately 40%) and progression (approximately 21%) 1. Currently, the risk of HGT1 progression cannot be estimated based on classic clinicopathological prognostic markers. Several histological subtypes are known to have poor prognostic outcomes in HGT1 management. A well-known example is the presence of a micropapillary variant associated with adverse pathological features and poor outcomes 2. The micropapillary subtype (diagnosed when there is a micropapillary component > 10%) represents approximately 0.6–2.2% of urothelial tumors 3-5.

Efforts have been made to establish a molecular classification for bladder cancer, particularly in the muscle-invasive stage 6,7. Data from these studies have been jointly reviewed to develop a consensus classification of the six subtypes of MIBC with different degrees of luminal and basal characteristics 8. More recently, molecular classification of NMIBC has been described for NMIBC 8-10. The large NMIBC analysis of the multi-institutional UROMOL study subclassified the tumors into four types 11,12, however, these subtypes are still not used clinically. One initial limitation is the extensive intratumor heterogeneity recently described with the advent of single-cell methodologies 13,14.

An important characteristic of bladder cancer across stages is the occurrence of recurring mutations, some of which affect genes involved in chromatin structure, such as KMT2A and KDM6A 15,16. We previously investigated the cancer cell-intrinsic genetics of HGT1 NMIBC by leveraging human patient samples 15,17. Our results showed a higher similarity in the pattern of somatic mutations between HGT1 and MIBC as compared to low-grade (LG) NMIBC 15,18,19. The genetic similarity between HGT1 and MIBC suggests the activation of invasive mechanisms in HGT1. Considering the high abundance of genetic alterations in chromatin genes at a similar level between MIBC and HGT1, we hypothesized that the mechanisms associated with progression could be impacted by chromatin structure 15,20. Some of the identified mutations in chromatin genes in HGT1 affect the genes involved in different levels of enhancer regulation. For instance, ARID1A, which is associated with disease progression in HGT1 15 is a member of the chromatin remodeling complex SWI/SNF 21. KMT2D methylates H3K4 at promoters and enhancers 22 and EP300 is a histone acetyltransferase involved in enhancer activation 16,23. This high representation of mutations in chromatin remodelers suggests an impact of chromatin reprogramming on BC tumorigenesis across stages.

Chromatin analysis, even in bulk, can reveal truncal lineages because chromatin states are less transient and reflect the regulatory potential more than the current gene expression levels 20,24. Based on this, we performed H3K27ac profiling of HGT1 tumors. This led to the discovery of three chromatin subclasses, two subclassifying classic URO histology, and a distinct one associated with the micropapillary (MP) histological subtype. The subtypes differ in their relevant molecular characteristics and are associated with the outcome. Single-cell, immunohistochemistry, and spatial transcriptional analyses showed that the two URO subtypes are not homogeneous across cancer areas but occupy distinct spatial regions. The differences between the histology of URO and MP are seen in a markedly distinct tumor microenvironment (TME). MP is characterized by increased stromal infiltration, enriched populations of cancer associated fibroblasts (CAFs), and predominance of M2-polarized macrophages interspersed within the tumor. Our findings highlight the complex heterogeneity of HGT1 bladder cancer and underscore the relevance of single-cell and spatial resolution analyses to advance target discovery and biomarker identification in precision medicine.

## Results

### Chromatin analysis reveals subtypes in HGT1 bladder cancer

**Figure 1:**
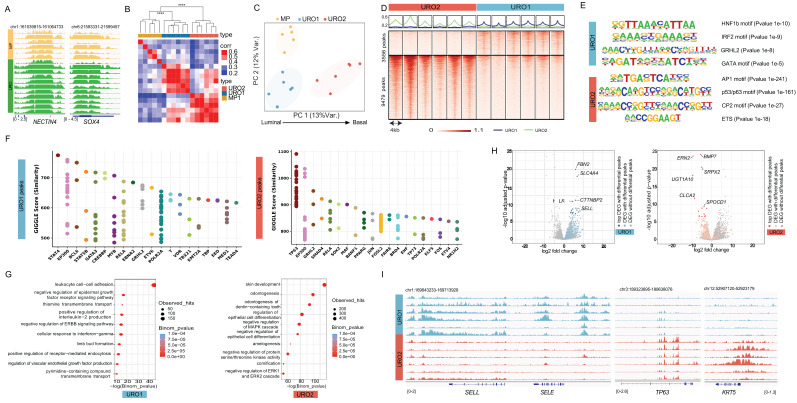
Epigenetic analysis of HGT1 tumors. A) Genome tracks of H3K27ac FiTAc-seq data from HGT1 samples at the NECTIN4 (scale 0-2.5 in number of reads per million per base pair (rbm) and SOX4 loci (scale in rbm) for MP subtype (top, in yellow) and URO subtype (bottom, in green). B) Heatmap showing the correlation between HGT1 samples. Above the heatmap is a hierarchical clustering of the samples indicating the statistical significance of the three main branches of the tree, Pvalue=0.001 (Permanova test). C) Principal component analysis (PCA) representing the same data showing the three clusters identified in the correlation analysis. PC1 illustrates the axis of Luminal to Basal characteristics. D) Heatmap representation of the differential H3K27ac regions distinguishing URO1 (n=3556 up peaks) and URO2 (n=9479 up peaks) samples. (scale in rbm) E) Top enriched motifs at the center of the differential peaks between URO1 (top) and URO2 (bottom). F) Similarity (GIGGLE) scores between regions of increased H3K27ac signal genome-wide in URO1 (left) and URO2 subtypes (right), and GEO-archived datasets of ChIP-seq for transcription factors. On the URO1 side, top overlapping TF is STAT4 in lymphocytes. For the URO2 peaks top overlapping is TP63 in keratinocytes. G) Gene Ontology analysis showing the enriched pathways in genes with nearby LLI specific H3K27ac regions (left) and BL specific H3K27ac regions, determined by GREAT analysis. H) Association between differential H3K27ac regions and differential gene expression for URO1 (left) and URO2 (right). Each volcano plot depicts RNAseq log2-fold change (x-axis) and p-value adjusted for multiple hypothesis testing (y-axis) as calculated by DESeq2. Each dot represents one gene with blue dots (left) indicating significant differential expressed genes (DEG) with a differential H3K27ac region nearby for the LLI subtype. Orange dots on the right represent the same for DEGs in the BL subtype. Gray dot: DEG with no significant differential H3K27ac region nearby. I) Representative IGV tracks at SELL and SELE showing super enhancers for the LLI subtype and TP63 and KRT5 (scales in rbm) superenhancer for the BL subtype.

We explored the status of activation of enhancers and super-enhancers (SE) by H3K27ac profiling of a cohort of 17 HGT1 patients with clinically annotated outcomes using FiTAc-seq analysis of FFPE archived clinical tissues, as previously described by our group 25 Table S1. We included a set of samples enriched for micropapillary (MP) content, which is known to be associated with worse clinical outcomes 26. For all cases included in the analysis, we ensured a minimum of 80% enrichment in cancer cells, performing macrodissection whenever required. The analysis produced high-quality results in terms of the identified peaks and the fraction of reads in the peaks (summarized in Table S2). The results showed activation of enhancers at previously known genes related to bladder cancer, such as NECTIN4 and SOX4 across the cases (Figure 1A), validating the specificity and quality of the results.

Unsupervised hierarchical analysis of the H3K27ac-marked (transcriptionally active) regulatory regions (promoters and enhancers) distinguished three statistically different clusters (Figure 1B). Two clusters subdivided the conventional urothelial histology (URO) and the other, corresponding to the MP histology (Figure 1B-C). Of the two URO clusters, URO1 was closer to the micropapillary (MP) than to the other URO clusters (URO2) (Figure 1C). Next, we performed a differential analysis 27 to identify regulatory sites specific to each of the three clusters Figure S1A). To investigate the transcriptional mechanisms underlying chromatin differences, we applied HOMER analysis (28 to assess the enrichment of transcription factor (TF) DNA-binding motifs in the differential regions. The results were concordant with the luminal origin of the MP cluster by showing enrichment of the GRHL2 motif in the differential regions of the MP cluster Figure S1B) (22,29. In contrast, the differential regions for the URO2 cluster showed TP63 as the top enriched motif Table S3, suggesting more basal-like characteristics for this cluster 30,31. Closer to the MP than to the URO2, the URO1 cluster showed enrichment in motifs for inflammation-related TFs, such as IRF2 and HNF1, supporting a more luminal-like inflammatory phenotype Table S3. In summary, chromatin analysis showed three distinct clusters that were positioned on an axis (PC1) from the luminal to the basal phenotype (Figure 1C).

### Urothelial tumors segregate into basal-like and luminal-like inflammatory chromatin states

We further investigated urothelial subtypes without the potential confounding parameter of MP histology, focusing on URO cases and assessing the differences between their two clusters. Comparison of URO1 with URO2 resulted in the identification of 3556 regulatory regions differentially H3K27ac- activated in URO1 and 9479 differentially activated in URO2 (Figure 1D), showing differences within the same histological subtype. Motif analysis of these two sets of regions showed similar results to those observed in the previous analysis, although it included enrichment in GATA motifs on the luminal-like side (Figure 1E). Next, we compared the differentially active regions to published chromatin immunoprecipitation sequencing (ChIP-seq) profiles compiled in CistromeDB 32. The results showed that the top URO1-peaks overlap with STAT4 and GATA3 peaks in previously published datasets. The overlap with these transcription factor binding sites validates the inflammatory and luminal phenotypes revealed by motif analysis (Figure 1F). The URO2-activated regions showed the highest overlap with TP63 binding in keratinocytes, validating the basal characteristics (Figure 1F). Consistent subtype characteristics were also exhibited when differential regions were analyzed using the Genomic Regions Enrichment of Annotations Tool (GREAT) analysis 33. This tool associates genomic regions with nearby genes and then examines the enrichment of Gene Ontology (GO) pathways for the set of genes associated with each specific subtype. The results showed that the immune pathway “Leukocyte cell-cell adhesion” as the top enriched pathway for the URO1 cluster (Figure 1G) while showing enrichment for “skin development” and “cornification” for the URO2 cluster (Figure 1G).

### Chromatin-derived subtype signatures integrate enhancer activity with transcriptional programs

Next, we integrated the differentially activated promoters and enhancers with gene expression from a subset of the 17 cases for which we had available RNA-seq 17 (Figure 1H) by combining the differential regions between URO1 and URO2 with the corresponding differential gene expression. As shown in the volcano plot, the top differentially expressed genes with corresponding differential enhancer activation in their vicinity included relevant genes involved in EMT and angiogenesis, such as *BMP7*, *ERN2*, and* SRPX2* 34,35 for the URO2 cluster (Figure 1H). The URO1 side showed a more complex phenotype, including a number of genes associated with immune pathways, such as *SLC4A4* 36,* KALRN* 37 and *SELL* 38 (Figure 1H). The relevance of the inflammatory genes for URO1 was further revealed by the presence of superenhancers (SE) at some of these genes, as is the case for *SELL* and *SELE* (Figure 1I), along with SE at luminal genes such as* GATA3* 39,40 Figure S1C). Conversely, URO2 showed activation of a transcriptional circuit for TP63 where motif enrichment was accompanied by activation of the super-enhancer at the *TP63* locus (Figure 1I, Figure S1C), potentially involved in the maintenance of the basal lineage for the URO2 cluster (41. The basal lineage was further reflected by the presence of an SE at *KRT5* (Figure 1I).

Overall, our findings revealed the presence of distinct chromatin subtypes in HGT1 bladder cancer samples. We showed that urothelial histology can be further divided into basal-like (BL) and luminal-like inflammatory (LLI) subtypes and that MP histology is associated with a distinct chromatin profile, in this limited cohort. While luminal and basal subtypes have been previously described in bladder cancer, our study established a chromatin-based framework for these subtypes and extended it to the MP histological subtype.

### Single-cell chromatin accessibility confirms subtype identity

Next, we assessed the homogeneity of chromatin subtypes in HGT1 by performing single-cell (sc) ATAC-seq analysis on two independent HGT1 patient samples from a separate cohort (HGT1_1 and HGT1_2). Using our previously established protocol 42 we isolated nuclei from frozen clinical tissue and performed scATAC sequencing with 10X Genomics. After filtering out low-quality nuclei, 12,173 nuclei were retained across both samples for analysis Figure S1D). Combined UMAP analysis of the two patient samples revealed multiple clusters (Figure S1D). To distinguish tumors from normal cell populations, we applied Copy Number Variation (CNV) inference (43,44 Figure S1E). Notably, while clusters of normal cells overlapped between the two patients, the clusters of cancer cells remained distinct, indicating greater chromatin structural differences in tumor cells than in normal cells (Figure S1D, 1F). To further characterize normal cell lineages, we leveraged an atlas of previously published scATAC datasets from normal human tissues (45. By projecting our samples onto the UMAP representation of this reference dataset, we identified a diverse set of normal cell types, including B cells, T cells, macrophages, endothelial cells, and smooth muscle cells Figure S1F).

The top genes with differentially activated enhancers and corresponding overexpression in the bulk analysis were used to generate two signatures, designed to classify cancer cells as either LLI or BL subtypes (Figure 1H) (from now on: Chromatin-derived score (CDS)). To further investigate the subtype distribution, we applied the gene activity score from this signature to scATAC-seq data (Figure S1G) (Table S1. The analysis revealed distinct differences between the two tumors: HGT1_1 exhibited stronger enrichment for the BL subtype, whereas HGT1_2 was more enriched for the LLI subtype Figure S1G).

Supporting these classifications, the subtypes were aligned using well-established lineage markers. BL-scored cancer cells showed increased *KRT5* promoter accessibility along with a higher enrichment of the TP63 motif (Figure S1H-I). Conversely, cells with lower BL scores exhibited increased *KRT20* promoter accessibility and GATA3 motif enrichment, consistent with an LLI phenotype (Figure S1H-I). Despite the overall homogeneity within each tumor, the alignment between the CDS scores and known markers was not complete. A subset of cells lacked accessibility to both keratin promoters, which is indicative of terminal differentiation states (Figure S1G). This incomplete overlap suggests a spectrum of differentiation within each subtype, with only a fraction of the cells expressing terminal markers. Such a continuum of differentiation states could also explain the varying enrichment of transcription factor motifs observed intratumorally across the clusters (Figure S1J). Overall, we concluded that the CDS classifier, derived from a chromatin-based signature, may provide robust lineage stratification independent of differentiation status.

### Expression of the LLI and BL chromatin derived subtypes in two independent urothelial bladder cancer patient cohorts

To assess the representation of the two URO subtypes, Basal-Like (BL) and Luminal-Like Inflammatory (LLI), in an expanded cohort of high-grade T1 (HGT1) urothelial tumors, we applied the Chromatin Differentiation Score (CDS) to score a bulk RNA-seq dataset of 62 previously collected and analyzed HGT1 cases (17 Table S1.

**Figure 2:**
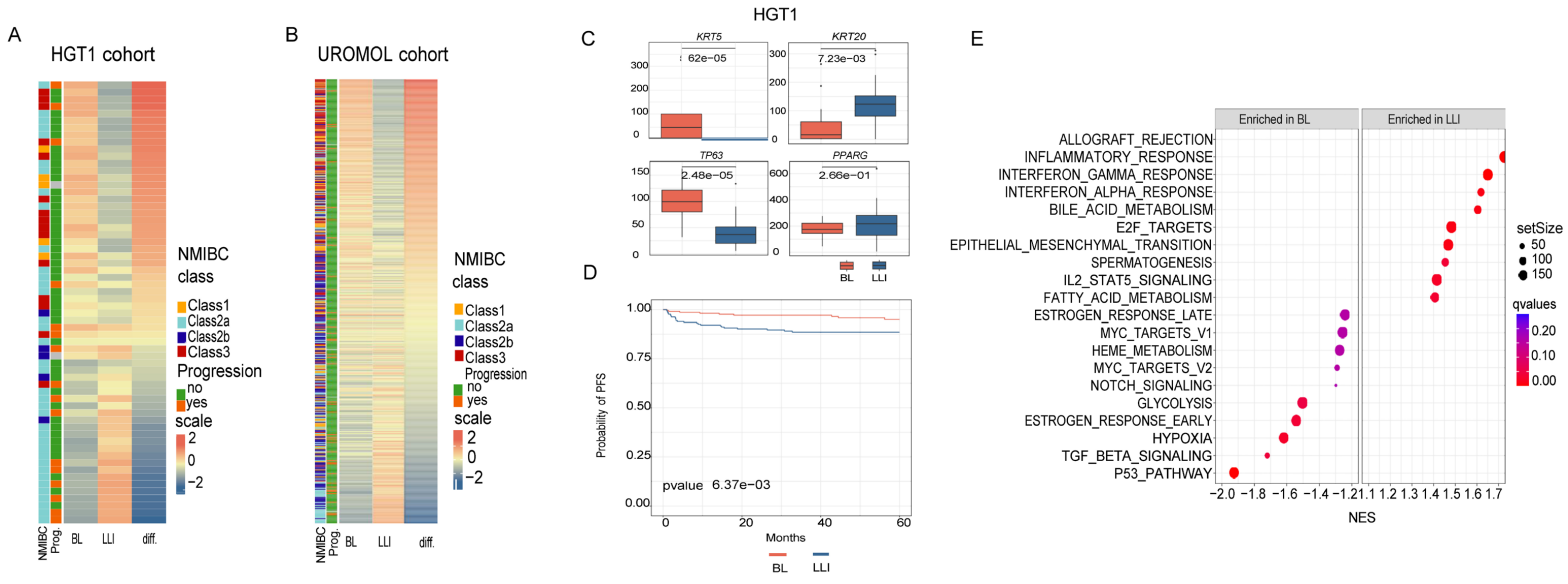
Analysis of chromatin-derived signatures in two NMIBC patient cohorts. A) Heatmap of BL and LLI signature scores analyzed from gene expression in HGT1 cohort (n=62). Patients are ordered from most BL to most LLI and also annotated by UROMOL2021 classification in the 4 subclasses (1, 2a, 2b and 3). The clinical outcome is also annotated for the cases as cancer progression (yes (orange) and no(green)). There is a statistically significant association between cancer progression and LLI subtype (p = 0.02). B) Heatmap of BL and LLI signature scores analyzed on the UROMOL gene expression cohort, ordered and annotated as in A. The clinical outcome is also annotated for the cases as cancer progression (yes and no). C) Boxplots showing the expression of marker genes analyzed in cases scored as BL (red) or LLI (blue) in the HGT1 cohort (difference analyzed by t-test). D) Kaplan-Meier survival curves for BL and LLI groups in the UROMOL cohort. Cases were dichotomized between BL and LLI based on the median of the score values. E) Gene Set Enrichment Analysis (GSEA) of BL and LLI differential genes in HGT1 cohort. The differential analysis was done comparing the top quartiles of score values (most BL vs most LLI).

The CDS results revealed a continuous distribution of scores ranging from cases with high BL signatures to those with high LLI signatures, with many cases exhibiting mixed characteristics (Figure 2A). A similar spectrum was observed in the UROMOL dataset, an independent cohort of 438 NMIBC cases, including 78 HGT1 tumors 11 (Figure 2B). In both cohorts, LLI cases exhibited significantly higher expression of KRT20, whereas BL cases were enriched for *KRT5* and *TP63* expression (Figure 2C). Interestingly, other luminal markers, such as *PPARG*, *GATA3*, and *CDH3* did not show subtype-specific expression patterns in either cohort (Figure 2C, Figure S2A-B), potentially because of lower expression levels and higher inter-patient variability compared to keratin genes.

To contextualize our classification, we compared our CDS-based subtypes with the molecular subtypes described by Lindskrog *et al*. (UROMOL2021), which define four NMIBC classes with varying basal and luminal features 11. Although our classification defines two distinct chromatin-defined subtypes, notable similarities have emerged. For instance, BL and UROMOL2021 Classes 1 and 3 shared elevated *TP63* expression, whereas LLI and UROMOL2021 Class 2a were both characterized by high *KRT20* and *ERBB2* expression (Figure 2A-B). These overlapping molecular features supported the existence of biologically distinct NMIBC subgroups with chromatin-associated differences.

We observed a significant overlap between the LLI subtype and UROMOL2021 Class 2a in the UROMOL cohort (chi-squared test, p=3.3E-08), while BL showed features like Class1 and showed significant overlap with Class 3 (chi-squared test, p<2.2 E-16). As UROMOL2021 Class 2a has been linked to an increased risk of disease progression 11 we next aimed to stratify UROMOL cases by CDS (BL vs. LLI) based on the median score to evaluate LLI and patient risk. Our results also revealed that the LLI subtype was significantly associated with a higher risk of progression (Figure 2D). This trend was also observed in the 62-HGT1 cohort, where LLI cases had a significantly increased likelihood of progression to muscle-invasive bladder cancer (MIBC) (p = 0.02; Figure 2A), despite the smaller sample size (Figure 2A).

To further explore the biological differences between the subtypes, we performed differential gene expression analysis between the top and bottom CDS quartiles in the 62-HGT1 cohort (Figure 2A), identifying 1,115 differentially expressed genes. Gene Set Enrichment Analysis (GSEA; Subramanian *et al*., 2005) revealed that LLI tumors were enriched in inflammatory pathways, including “Allograft rejection,” while BL tumors were enriched for the “p53 pathway”, “TGF_BETA signaling, and “Hypoxia” (Figure 2E).

Taken together, these findings confirmed the existence of two distinct chromatin states, BL and LLI, in clinical HGT1 bladder cancer samples from two independent cohorts. The results validated their associated transcriptional programs and showed that the LLI subtype is associated with poorer clinical outcomes.

### Single cell expression analysis validates the existence of subtypes and shows intratumor heterogeneity

To assess heterogeneity at single-cell resolution in a larger cohort, we performed single-nuclei RNA sequencing (snRNA-seq) using GEM-X Flex technology from 10XGenomics, on nine FFPE tumors from the 62-HGT1 cohort, along with two additional MIBC surgical specimens. These latter tumors were selected based on their combined micropapillary >30% and URO histology (hereafter referred to as MPBC1 and MPBC2). Nuclei were isolated using an in-house FFPE-optimized protocol, which builds on our previously published method for frozen tissue 42 and includes additional deparaffinization and rehydration steps.

This approach yielded 37,879 high-quality nuclei in 11 patients Table S1. Data were visualized using UMAP to examine inter- and intratumoral heterogeneity Figure S3A). Malignant and nonmalignant populations were distinguished using a combination of CNV inference via inferCNV (46 and the expression of cancer markers, such as *NECTIN4* Figure S3B-C). Consistent with our scATAC-seq findings (Figure S1D), snRNA-seq shows that normal cell clusters were more homogeneous across patients, whereas tumor cell clusters displayed higher heterogeneity (Figure S3A,D). Lineage annotation based on canonical markers (as described in Methods), identified diverse normal cell types including T cells, B cells, endothelial cells, fibroblasts, plasma cells, and normal epithelial cells (Figure S3D-E). These findings confirm the ability of our FFPE snRNA-seq workflow to preserve the TME complexity.

**Figure 3:**
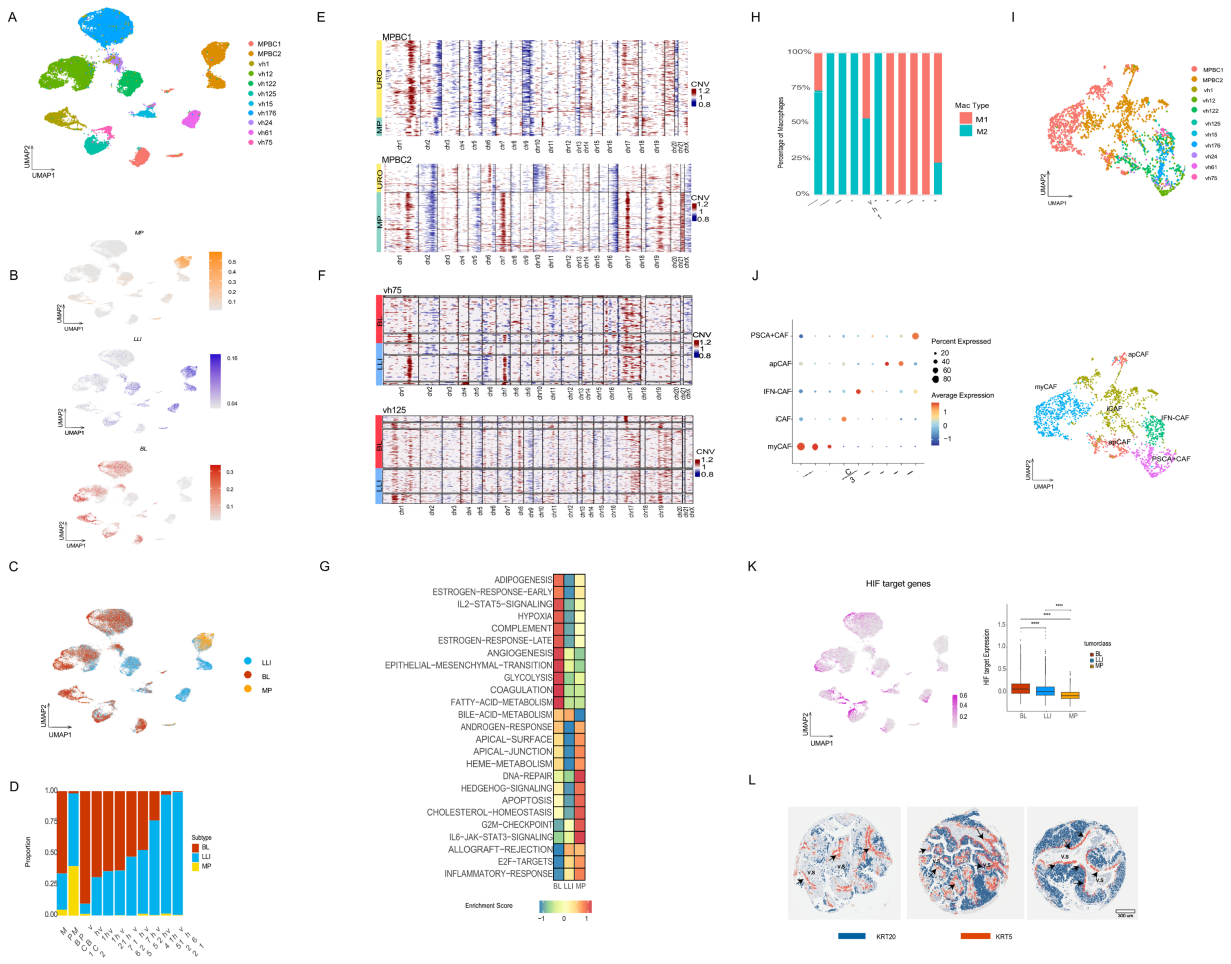
snRNA-seq study of nine HGT1 cases and two MIBC with mixed URO and MP histology. A) UMAP combining the cancer cells from the eleven datasets, colored by sample ID. B) UMAP plot showing the scoring of all the cancer cells in our study by the CDS classificator in MP, LLI and BL chromatin derived signatures (scale on the right, represents enrichment score values). C) CDS classification of the cancer cells in the combined UMAP plot showing cancer cells scoring for the LLI subtype (blue), the BL (red) and the MP subtype (yellow). D) Barplot showing the relative percentage of each cancer subtype in each tumor sample. E) CNV analysis of the cancer cells in the two micropapillary mixed samples clustered by histological subtype. These cell populations show distinct CNV patterns indicating that they are distinct clones. The MP clones from both MPBC samples show alterations on chromosome 17 consistent with ERBB2 amplifications. The color scale represents relative copy-number variation (CNV) signal. Values are centered around 1 (neutral copy number). Red indicates copy-number gains, while blue indicates copy-number losses. F) CNV analysis of two representative URO samples (vh75 and vh125) separated by CDS subtype and then clustered with Kmeans=3 to show the subclonal structure. In both cases the BL components of the tumor show similar clonal structure as the LLI cells. The scale is as in panel E. G) GSEA pathway analysis of the differentially expressed genes for BL, LLI and MP pseudoclusters of cancer cells. H) Bar plot showing the relative percentage of M1 and M2 macrophages in each tumor sample. I) UMAP of all CAFs identified across the eleven datasets and colored by sample ID. J) Subclassification of the CAF population based on the expression of canonical markers into the following subtypes: myCAF, iCAF, apCAF, INF-CAF, and PSCA-CAF. Left: Dot plot showing marker gene expression across annotated cell types. Dot size represents the percentage of cells within each cell type expressing the gene, and color intensity indicates the average scaled expression level. Canonical marker genes were selected to support cell-type annotation. Right: UMAP of the subclassified CAF populations from panel I, showing that myCAFs are present only in the two MPBC samples. K) UMAP plot showing the scoring of all the tumor cells in our study with a HIF target genes signature. Right bar plot shows the quantification of the HIF target gene signature across the subtypes for the entire snRNA-seq cohort. L) Three representative NMIBC cases from a TMA that are positive for KRT5 and KRT20 staining. The two stains have been merged showing that the positive cell populations are nearly mutually exclusive. Also, the KRT5+ cells (orange) are largely near the blood vessels (v.s.) with the KRT20+ cells (blue) further away.

Focusing on malignant cells (Figure 3A), we evaluated the cancer subtypes. To this end, we extended the CDS framework to include a MP-specific signature, derived analogously to the BL and LLI signatures, by identifying MP-specific enhancers and corresponding gene expression (Figure S3F). We then performed subtype scoring using CDS, classifying cells as BL, LLI, or MP (Figure 3B-C). We observed varying degrees of intratumoral heterogeneity, with BL and LLI cancer cells coexisting within individual tumors, particularly among URO carcinomas (Figure 3C-D). As expected, the MP signature showed the highest scores in MPBC cases with a mixed MP/URO histology, validating its applicability in these independent cases (Figure 3C-D). In these instances, the URO component of MPBC1 was scored mainly as the BL subtype, whereas in MPBC2, the URO component was classified as LLI (Figure 3D). These findings further suggest that HGT1-derived signatures may also be applicable to MIBC tumors.

Interestingly, a few tumors diagnosed with pure URO histology, such as vh1, vh24, and vh122, contained small cell populations with elevated MP scores, suggesting previously unrecognized MP histology (Figure 3D). Among the URO cases, several tumors (vh12, vh176, vh125, vh75, vh24, and vh15) exhibited a mixture of BL and LLI cells, whereas vh1 and vh61 were predominantly BL and LLI, respectively (Figure 3D). Tumor vh122, although classified as LLI, lacked KRT20 expression (Figure S3G) and harbored an MSH2 splice-site mutation (15 which may enhance neoantigen production and drive inflammation (Figure 3C-D). In accordance with their lineage characteristics, the snRNA-seq results also showed that the BL subtype was associated with *KRT5* and *TP63*, and LLI/MP with *KRT20* Figure S3G).

Next, we compared CNVs profiles across subtypes. In the two mixed-histology cases (MPBC1 and MPBC2), the MP and URO components harbored distinct clonal profiles showing two distinct genetic backgrounds (Figure 3E). In contrast, within URO histology, the BL and LLI subtypes shared similar clonal origins (Figure 3F), suggesting epigenetic regulation rather than genetic divergence. Distinct chromatin subtypes with the same genetic characteristics support the occurrence of cancer cell plasticity in URO histology, based on chromatin switching. In contrast, the distinct genetic structures of MP and URO histologies suggest more stable subtypes.

To benchmark our classification strategy, we compared CDS scores with previously proposed UROMOL2021 subtypes (11. When applying the UROMOL2021 classification to the nine HGT1 snRNA-seq datasets, we observed the coexistence of multiple subtypes within individual tumors; for example, vh125 and vh176 showed a mixture of classes 2a and 3, consistent with previously reported intratumoral heterogeneity 47 Figure S3H). Notably, we could not identify cells classified as class 2b in our single-cell analysis (Figure S3H). This finding suggests that class 2b may be the result of a mixed phenotype at the bulk level rather than a distinct tumor entity.

We then used pseudo-bulk clustering and single-sample GSEA to identify the pathway enrichments per cancer subtype (Figure 3G). BL clusters were enriched in “angiogenesis” and “hypoxia” pathways; LLI clusters in “inflammatory” and “allograft rejection” pathways and MP clusters in metabolism related like “cholesterol-homeostasis” and in cell polarity like “apical junction” pathways (Figure 3G). These pathways involve the interaction between cancer cells and the microenvironment, which we can further investigate using single-cell methodologies.

### Assessment of subtypes and microenvironment by single-cell transcriptional analysis

When analyzing the TME, we observed several notable features. One consistent finding across all subtypes was the presence of M2 macrophage polarization, as determined using canonical markers including CD163 (Figure 3H) (described in the Methods section). In particular, MP tumors demonstrated activation of mucins, such as MUC4 (Figure S3F), which is involved in glycosylation and is known to contribute to a pro-tumorigenic immune environment. For instance, the SPP1–MUC4 axis has been implicated in promoting M2 polarization (48 49. Consistently, the two MPBC samples with MP components showed a higher abundance of M2-polarized macrophages as compared with the URO cases (Figure 3H). However, this is a very small cohort, and the results would need to be validated in larger cohorts. The M2 polarization observed in general in bladder cancer suggests a pervasive subtype-independent immunosuppressive microenvironment.

Next, we analyzed the status of Cancer-Associated Fibroblasts (CAFs), which are known to promote M2 macrophage polarization 50. CAFs can be categorized into different subtypes including: Myofibroblastic CAFs (myCAFs), immunoregulatory inflammatory CAFs (iCAFs), Antigen-Presenting CAFs (apCAFs), IFN dependent CAF (IFN-CAFs), and Prostate Stem Cell Antigen (PSCA)+ CAFs (PSCA+CAFs) 51. Previous studies used inferred CAF signatures from the UROMOL bulk RNA-seq cohort-associated UROMOL2021 class 2b with high fibroblast signature activity and class 3 with immune depletion and low CAF activity 52. However, these subtypes do not appear to represent distinct entities when analyzed using single-cell approaches, and the characteristics of CAFs in bladder cancer remain poorly defined.

Our single-cell resolution data provides new insights into this question. The CAF were reclustered (Figure 3I) showing presence of the distinct CAF subtypes in the cohort, as assessed by the expression of specific markers (Figure 3I, J). Both MPBC samples exhibited myCAF populations, with MPBC2 showing a mixture of myCAFs and iCAFs (Figure 3J). Both MPBC samples showed a very low percentage of IFN-CAFs and PSCA^+^ CAF (lower than 3% combined for these subtypes in both cases) (Figure 3I, J). The HGT1 cases, conversely, were enriched in INF-CAFs and PSCA^+^ CAF. Notably, VH122, which harbors an MSH2 mutation, was enriched in apCAFs (Figure 3I, J), suggesting a potential role for apCAFs in neoantigen presentation as a result of MSH2 mutation.

Within the URO histology group, we observed notable differences in pathway enrichment between subtypes (Figure 3G). Interestingly, “hypoxia” and “angiogenesis” pathways were significantly enriched in the BL subtype compared to LLI (Figure 3G), with marked differential activation of the HIF1 target pathway in BL tumors (Figure 3K). These findings suggest that the chromatin-defined subtypes are associated with distinct microenvironmental conditions.

To spatially validate the intratumor heterogeneity in the expression of cancer subtypes and its spatial compatibility with the enriched pathways, we performed immunohistochemistry (IHC) on tissue microarrays (TMAs) from an independent cohort of 162 pure urothelial tumors 53 comprising 102 high-grade T1 (HGT1), 41 low-grade, and 19 muscle-invasive bladder cancer (MIBC) samples. We applied immunostaining of KRT5, TP63, and KRT20 as surrogate markers for BL and LLI subtypes. Notably, coexistence of KRT5- and KRT20-expressing tumor cell populations was observed in 44% of the samples (53/120), reflecting intratumoral heterogeneity Figure S3I). In contrast, exclusive expression of KRT5 (27%), KRT20 (21%), or neither marker (8%) further underscored subtype diversity, patterns consistent with those observed in our scRNA-seq dataset (Figure 2A,B).

Spatial mapping confirmed subtype-specific cellular organization: KRT5 positive cells were enriched at the tumor–stroma interface, whereas KRT20 positive cells were centrally located (Figure S3J). TP63 primarily overlapped with KRT5, indicating a distinct BL subset. Minimal co-expression supported the statistical spatial separation of the subtypes (Figure S3K, 3L). The localization of basal KRT5 positive cells was significantly closer to the vascular stroma (Figure S3J,K) supports the idea that oxygen availability may play a role in maintaining BL and LLI phenotypes, with BL cells showing increased sensitivity to hypoxic signaling.

Together, these findings underscore the value of single-cell analysis combined with IHC to resolve the molecular and spatial complexity of bladder cancer. Both methods highlighted the coexistence of transcriptionally distinct subtypes within individual tumors and emphasized the biological relevance of spatial organization for understanding tumor heterogeneity and progression.

### Assessment of subtypes and microenvironment by spatial whole transcriptional analysis

**Figure 4:**
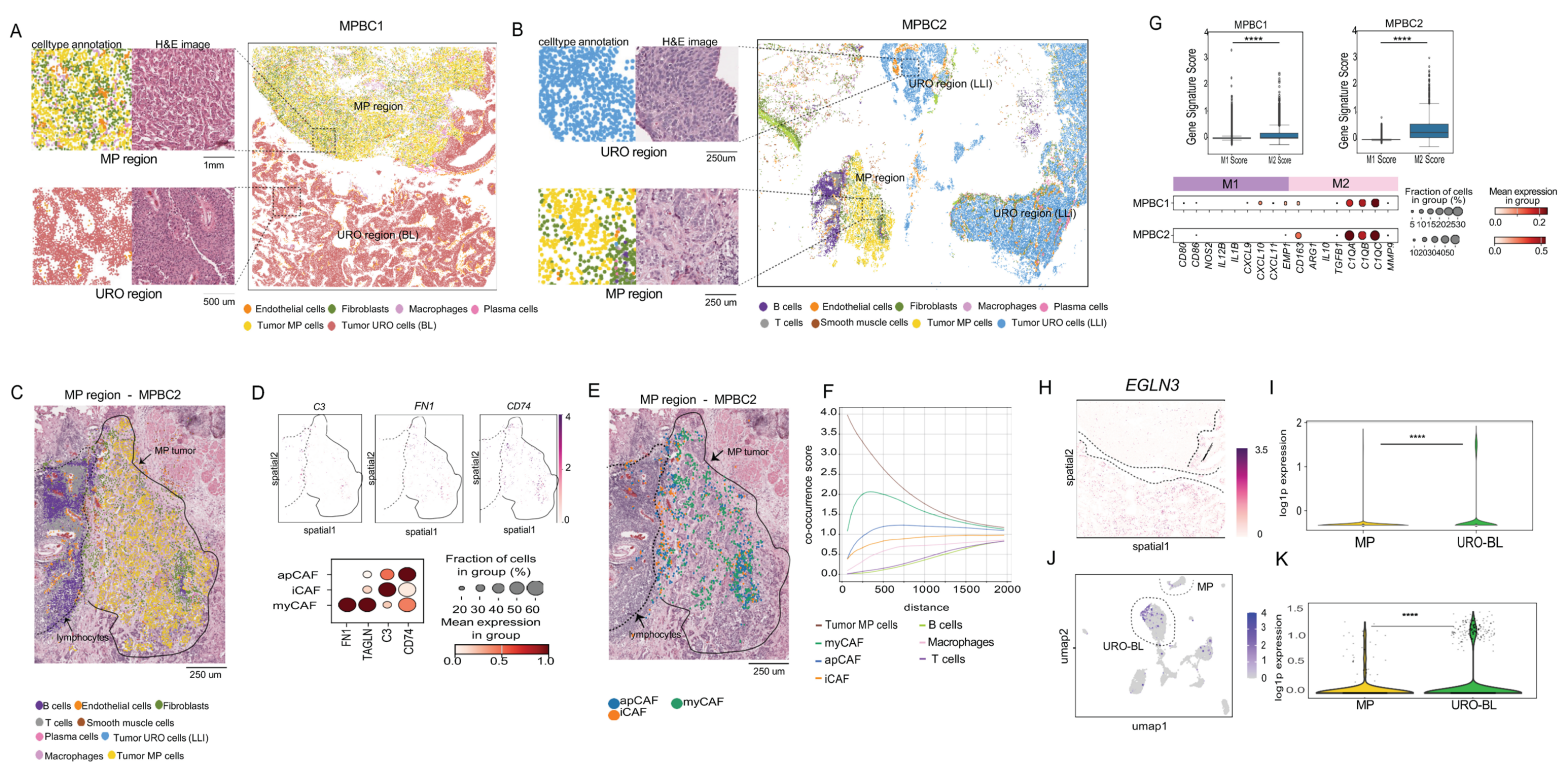
Spatial transcriptomic analysis of two MIBC with mixed URO and MP histology. A) Spatial transcriptomic results from MPBC1. Cells are colored by cell type. Dashed boxes indicate regions expanded to the left of the main figure, where the H&E image and cell types are shown in representative MP and URO regions. The MP component shows a highly infiltrated TME, where cancer cells represent 49% of total cells in the area. In the URO component of MPBC1, cancer cells represent the 96% total cells in that area. B) Spatial transcriptomic results from MPBC2, as shown in part A. In the MP component of MPBC2, cancer cells represent 58% of the total cells in the area, compared with 77% cancer cells in the URO region. C) Expanded view of the MP region of MPBC2 showing cell type annotation on top of the H&E image. The solid line surrounds the MP tumor cells and the dashed line demarcates the immune cell cluster. D) Analysis of the fibroblast population in this region using mean expression of canonical markers across a high fraction of cells to identify the CAF subtypes (apCAF, iCAF, myCAF). Dot plot shows CAF- specific marker gene expression across annotated cell types. Dot size represents the percentage of cells within each cell type expressing the gene, and color intensity indicates the mean expression level. E) Expanded view of the MP region of MPBC2 showing CAF subtype annotation on top of the H&E image. The solid line surrounds the MP tumor cells and the dashed line demarcates the immune cell cluster. F) Quantitative analysis of the co-occurrence of CAF subtypes relative to the micropapillary tumor cells. The myCAFs are most closely associated with the MP population, showing the highest co-occurrence score across all distances, relative to the other subtypes. apCAFs are more towards the edges of the tumor region with an intermediate co-occurrence score while iCAFs have the lowest co-occurrence being almost completely excluded from the tumor regions and closer to the immune cell spatial clusters in MPBC2. The x-axis shows distance measured in pixel units. G) Spatial representation of EGLN3 target gene expression in the basal urothelial cells of sample MPBC1. Dot plot shows Macrophages- specific marker gene expression across annotated cell types. Dot size represents the percentage of cells within each cell type expressing the gene, and color intensity indicates the mean expression level. H) Spatial representation of EGLN3 expression in MPBC1, showing higher expression in the URO component, which in this case is scored as BL subtype. I) Violin plot of EGLN3 gene expression levels in MP cells versus the URO (BL) cells in the MPBC1 spatial dataset. Significantly higher expression is detected in the URO-BL cells (t-test; p<0.01). J) UMAP of EGLN3 expression levels in the MPBC1 snRNA-seq dataset, showing higher expression in the URO subtype compared with the MP component. K) Violin plot of EGLN3 expression levels in MP cells versus the URO (BL) cells in the MPBC1 snRNA-seq dataset. Significantly higher expression is observed in the URO-BL cells (t-test; p<0.01).

Next, we applied the 10x Genomics Visium HD platform to spatially investigate the transcriptional profiles of bladder cancer subtypes and to visualize their interactions with the TME. This technology enables transcriptome-wide spatial profiling at an enhanced resolution (2 × 2 µm contiguous barcoded squares) and integrates hematoxylin-stained histological imaging, enabling spatial mapping of transcription between cancer cells and their surrounding stroma. We conducted this analysis on two MPBC cases for which we had surgical specimens containing both micropapillary (MP) and urothelial (URO) histology (Table S1. Unlike biopsies, these samples preserved the tissue architecture required for spatial transcriptomic analysis (Figure 4AB; Figure S4A). We performed cell segmentation to assign transcriptional information to each cell using a bin-to-cell methodology that combines the H&E image with the transcriptional information, as described in the Methods section. Next, we performed clustering analysis using a graph-based method using the Scanpy/Squidpy framework 54,55 (Figure 4A-B and Figure S4B).

Unsupervised clustering revealed distinct clusters corresponding to normal cell lineages and cancer cell subtypes, which we annotated using a marker-based approach, as done for the snRNA-seq analysis and described in the Methods (Figure 4A-B; Figure S4B). Notably, the MP and URO histologies formed transcriptionally distinct clusters in the UMAP plot Figure S4B), which spatially matched their corresponding histological spatial locations as shown by comparison with the Hematoxylin-Eosin staining (Figure 4A-B). In MPBC1, the URO component scored as the BL subtype without significant LLI enrichment (Figure S4C), differing from the snRNA-seq results for the same tumor that also showed a LLI component (Figure 3D). This difference could likely be due to sampling from different tissue sections (Figure S4C). Conversely, the URO component of MPBC2 consistently scored as LLI subtype across both platforms (Figure S4D).

The spatial organization of the TME varied significantly between the MP and URO components. The URO-associated stroma displayed a relatively simple structure, whereas the MP histology featured a more complex stromal architecture with a lower representation of cancer cells versus stromal cells and a higher presence of cancer-intermixed macrophages and CAFs (Figure 4A-B).

Since our snRNA-seq analysis revealed the presence of myCAFs, iCAF, and apCAFs populations in MP samples, we aimed to investigate the spatial location of these CAFs subtypes. We found a particularly complex CAF representation in the MP component of MPBC2 (Figure 4C), that in accordance with the snRNA-seq result, showed significant representation of myCAFs, iCAF, and apCAFs subtypes based on the expression of corresponding canonical markers (Figure 4D). These three CAF subtypes in MPBC2, occupied distinct spatial niches: myCAFs were embedded within the tumor core, whereas iCAFs were localized to the periphery and were interspersed within the cluster of immune cells, including B cells, T cells, and macrophages (Figure 4E). We also found populations of apCAFs closer to the periphery of the tumor (Figure 4E). We further analyzed the co-occurrence probability of CAF populations relative to that of MP cancer cells. These results validated the spatial observation, showing that myCAFs were more likely to co-occur closer to MP cancer cells (Figure 4F). In MPBC1 in contrast, we did not find iCAF, but there was still a high population of myCAFs and apCAFs that were intermixed with MP cancer cells (Figure S4E-F). This result emphasizes the additional value of spatial technology in spatially contextualizing snRNA-seq findings.

The representation of M2-polarized macrophages was observed across all bladder cancer samples analyzed by snRNA-seq (Figure 3H). For MPBC1 and MPBC2 in particular, we found a statistically significant higher M2-to-M1 macrophage ratios (Figure 4G), as reflected by the expression of M2-associated markers (Figure 4G).

To investigate the TME in URO subtypes and the results from the IHC analysis, we spatially analyzed the oxygen-related features enriched in BL subtypes. The snRNA-seq results showed an enriched expression of “hypoxia” and “angiogenesis” pathways in the BL subtype compared to the LLI and MP (Figure 3G). In addition, immunohistochemical analysis showed that the KRT5 positive basal cells were significantly closer to the vascular stroma (Figure 3K). Therefore, we analyzed the spatial distribution of the standard hypoxia marker *EGLN3* within the BL component of MPBC1. *EGLN3* is a marker of sustained or late-stage hypoxic responses and is more stable than HIF1A mRNA, which can be transient (56. We observed strong overexpression of *EGLN3* in the BL component as compared with MP histology (Figure 4H-I), which was also validated in the snRNA-seq analysis of the same sample (Figure 4J-K). This result was extended to the snRNAseq 11 sample cohort, which showed that *EGLN3* was significantly higher in BL than in LLI and MP Figure S4G). These findings support that BL cells might exhibit a distinct sensitivity to oxygen availability, which is consistent with observations from immunohistochemical analyses. However, a comparison with the LLI component was not possible because of its absence.

In summary, spatial transcriptomics complemented and spatially contextualized snRNA-seq analysis, highlighting the complexity of the TME and revealing distinct differences between MP and URO histologies within the same tumor specimens.

## Discussion

### Chromatin profiling refines and validates molecular subtypes

In this study, we defined chromatin subtypes in HGT1 NMIBC based on enhancer profiling that show intratumor heterogeneity, that are associated with the activation of distinct molecular pathways and have divergent interactions with the TME. Previous classifications, using bulk RNA-seq lacked resolution to uncover this complexity (11, underestimate the significance of intratumor heterogeneity and limit the ability to correlate molecular subtypes with patients’ clinical outcomes. More recently, single-cell technologies have revealed the coexistence of subtypes within individual tumors 47, however, further refinement and functional interpretation of these subtypes are required. Our study builds upon these findings by anchoring subtype distinctions to the chromatin landscape. Specifically, our results provide chromatin support for the UROMOL2021 subtypes 1, 2a, and 3. By contrast, LC subtype 2b 11 may represent a heterogeneous mixture of cellular states rather than a discrete, transcriptionally, and epigenetically defined entity. This is supported by the lack of alignment between subtype 2b and any chromatin-defined subtype as well as the absence of 2b signature scoring at the single nucleus level in our cohort.

Our epigenetic profiling subdivides URO tumors into two distinct chromatin subtypes originating from potentially distinct cells of origin. Although we cannot rule out the existence of additional subgroups, especially given the size limitation of our H3K27ac dataset, we show the presence of two major states: a luminal-like (LLI) and a basal-like (BL) chromatin program, sharing features with UROMOL2021 subtypes 2a and 3, respectively. Therefore, our chromatin findings provide a mechanistic substrate that supports the stability and steady state of those UROMOL2021 subtypes. Chromatin states capture regulatory potential and lineage commitment that are less sensitive to transient transcriptional fluctuations than mRNA profiles alone. It also reflects the cell-of-origin either luminal or basal, that still shows chromatin characteristics and memory from lineage. Our work validates the existence of steady states subtypes 2a and 3 likely reflecting the lineage's determination.

### Epigenetic subtypes reflect tumor plasticity and spatial organization

The LLI and BL states were found to coexist in approximately 40% of the tumors analyzed. Copy number variation (CNV) inference from snRNA-seq data revealed that both URO subtypes share similar genetic backgrounds within the same tumor. Immunohistochemical analysis further supported this coexistence of the classification, showing that KRT5-positive basal-like cancer cells were frequently located near the vascularized stroma, whereas KRT20-positive luminal-like cells tended to cluster toward the tumor center. Taken together, the non-genetic mechanism, spatial compartmentalization, and variable degrees of heterogeneity across cases suggest that epigenetic subtypes may reflect tumor plasticity driven by environmental factors. For example, oxygen accessibility has been implicated in cancer plasticity 57 particularly through the oxygen-sensing activity of the H3K27 histone demethylase KDM6A. KDM6A (UTX) catalytic activity falls sharply as oxygen drops, leading to increased H3K27me3 at specific loci, impairing demethylation, and blocking differentiation. This drop in KDM6A activity allows cells to translate hypoxia into stable chromatin and transcriptional changes 57. Through this chromatin reprogramming, KDM6A could link oxygen availability to control of cell fate decisions and acts as a tumor suppressor whose inactivation can support adaptive responses in low-oxygen environments 58. Inactivating mutations in KDM6A, which are more common in bladder tumors with luminal features, may further support the role of hypoxia-mediated epigenetic reprogramming in shaping tumor heterogeneity. Further analysis of KDM6A mutational status and their correlation with predominant chromatin subtypes could further support this hypothesis.

### URO and MP subtypes display contrasting TME architectures

In contrast, the two distinct histological subtypes, URO and MP, which can coexist within the same tumor specimen, represent, in the studied samples, separate genetic clones and may correspond to more stable transcriptional states. Importantly, our analysis shows that URO and MP exhibit markedly different interactions between cancer cells and their respective TMEs. The MP subtype is characterized by a more complex microenvironment, with a diverse population of cancer-associated fibroblasts (CAFs) occupying spatially distinct regions. In the MP compartment of the studied cases, myofibroblastic CAFs (myCAFs) are found in close proximity to tumor cells, whereas inflammatory CAFs (iCAFs) are more intermingled with immune cells. This spatial organization coincides with higher infiltration of M2-polarized tumor-associated macrophages (TAMs) within the MP component. The presence of M2 macrophages and these CAF subtypes suggests an immunosuppressive microenvironment associated with the MP cellular niche. CAFs are increasingly being recognized as key regulators of tumor progression through mechanisms such as extracellular matrix remodeling, secretion of growth factors, and modulation of immune responses 51,59,60. Similarly, M2-polarized TAMs promote tumor progression and immune evasion by supporting tissue repair, angiogenesis, and immunosuppression 61. CAFs can recruit and polarize macrophages toward the M2 phenotype 62 via cytokine and fibroblast growth factors 63. The reciprocal interaction between CAFs and M2 macrophages reinforces an immunosuppressive TME and contributes to the resistance to immune checkpoint inhibitors in multiple cancer types 64. Specific TME characteristics can contribute to worse outcomes associated with MP histology. In contrast, the URO subtype exhibited an immune-excluded phenotype with fewer infiltrating immune cells, reflecting a less complex stromal landscape. Further studies in larger, independent cohorts will be required to assess the robustness and generalizability of these observed differences between histological subtypes.

### Summary

The presence of chromatin-based subtypes exhibiting intratumor heterogeneity and phenotypic plasticity underscores the complex challenges faced in cancer treatment. These potentially dynamic epigenetic states may enable tumors to adapt to therapeutic pressures, contributing to resistance and disease progression. Furthermore, the differences observed between the micropapillary (MP) and urothelial (URO) subtypes are based on variations in the TME, suggesting that microenvironmental factors may actively drive or sustain divergent tumor programs. Together, these insights highlight the complex heterogeneity of bladder cancer and underscore the relevance of single-cell and spatial resolution analyses in uncovering disease biology, ultimately guiding the development of more precise and context-specific therapeutic strategies.

## Methods

### NMIBC tissues and FiTAc H3K27Ac profiling

NMIBC tissues were obtained from collections at the Hospital del Mar-Parc de Salut MarBiobank in Barcelona, Spain. For H3K27ac profiling, we selected 17 NMIBC, five of which had identified micropapillary content Table S1. All the patients were treatment-naive. To increase the enrichment of cancer cells, we macro-dissected FFPE sections whenever needed to obtain >80% tumor cells. To increase the enrichment in micropapillary content, cores were taken at areas of high MP content, as selected by a pathologist. All procedures performed in studies involving human participants were in accordance with the ethical standards of the institutional and/or national research committee and with the 1964 Helsinki Declaration and its later amendments or comparable ethical standards. The FiTAc-seq method was performed as previously described 25. We started from 10 sections, 10 μm thick, that were washed three times with xylene to remove paraffin, and rehydrated in an ethanol/water series. The tissue was resuspended in lysis buffer as previously described and sonicated for 5 minutes using a Covaris E220 instrument (setting: 140 peak incident power, 5% duty factor, and 200 cycles per burst) in 1 ml adaptive focused acoustics (AFA) fiber millitubes. Soluble chromatin (5 μg) was immunoprecipitated with 10 μg of H3K27ac (Diagenode catalog number C15410196) antibody (Ab). ChIP-seq libraries were constructed using ThruPLEX-FD kits (Rubicon Genomics), following the manufacturer’s protocol. 75-bp single-end reads were sequenced using a NextSeq instrument (Illumina).

### Nuclei preparation and single-cell ATACseq

For each of the two HGT1 cases, a 40 μm section was obtained, and nuclear isolation was performed as previously described^25^,^42^ Briefly, the section was suspended in 300 μl of cold buffer comprising 0.1% NP40, 0.1% Tween-20, and 0.01% digitonin. The homogenized tissue was then transferred to a pre-chilled 1.5 ml microfuge tube and incubated on ice for 10 min. Lysates were then filtered through a 40 μm cell strainer, and nuclei were centrifuged for 10 min at 1500 relative centrifugal force (RCF) in a pre-chilled (4 °C) fixed-angle centrifuge. Nuclei were resuspended in 300 μl of buffer containing 0.1% Tween-20 and enumerated using a hemocytometer with trypan blue stain. Approximately 7000 cells were targeted per sample and processed following the 10× Genomics scATAC-seq sample preparation protocol (Chromium Single Cell ATAC Library & Gel Bead Kit, 10× Genomics). Paired-end reads of 150 bp were sequenced using NovaSeq XP.

### Nuclei isolation and snRNA-seq from FFPE tissue

Nine NMIBC clinical samples were selected from the Hospital del Mar-Parc de Salut MarBiobank in Barcelona, Spain. Two MPBC samples were obtained from the Gelb Center, Dana Farber Cancer Institute. Nuclei were isolated using a modified version of a protocol originally developed for frozen specimens, optimized for FFPE materials. Paraffin was removed through sequential xylene washes, followed by tissue rehydration in a graded ethanol series, and finally in distilled water. After centrifugation, the tissue pellet was resuspended in a dissociation buffer composed of phosphate-buffered saline (PBS, 896.5 µL), Liberase TM (38.5 µL at 6.5 U/µL), Collagenase D (40 µL at 100 mg/mL), and RNase inhibitor (25 µL at 40 U/µL). Samples were incubated at 37°C for 1 h in a thermomixer to facilitate enzymatic tissue dissociation. Following dissociation, the samples were washed with PBS, centrifuged, and the resulting pellet was resuspended in 300 µL of lysis buffer containing 0.1% NP-40, 0.1% Tween-20, and 0.01% digitonin. The suspension was incubated overnight at 37°C in a thermomixer to ensure complete cell lysis and nuclear release. After incubation, 700 µL of 0.1% Tween-20 RSB buffer was added before passing through a 40 µm cell strainer to remove debris. The nuclei were counted on Countess II before pelleting them down by centrifugation at 1,250 × g for 10 min at 4°C using a fixed-angle rotor. Approximately 10,000 nuclei per sample were processed for single-nucleus RNA sequencing using the 10x Genomics GEM-X Flex Gene Expression protocol. Libraries were prepared, multiplexed, and sequenced as 150 bp paired-end reads on the Illumina NovaSeq XP platform.

### Visium HD protocol

Spatial transcriptomic profiling was performed using the 10x Genomics Visium HD platform following the manufacturer's protocol with minor modifications. Formalin-fixed, paraffin-embedded (FFPE) tissue blocks were sectioned at 5 μm thickness and mounted onto standard glass slides. Sections were then fixed, stained with hematoxylin and eosin (H&E), and imaged using a high-resolution slide scanner to capture the detailed tissue morphology. Following imaging, tissue sections were permeabilized to facilitate probe access. Subsequently, probe hybridization was carried out overnight to target mRNA sequences.

After hybridization, the slides were processed using the Visium CytAssist instrument. This step involved aligning the tissue sections on standard glass slides with the capture areas on the Visium HD slide, enabling the transfer of spatially barcoded probes to the capture areas. The CytAssist instrument facilitates the transfer of spatial information from the tissue sections to the Visium HD slide. Library preparation was completed using the Visium HD library construction kit, and sequencing was performed on an Illumina NovaSeq system to a depth sufficient for highresolution spatial gene expression analysis.

### Immunohistochemistry

We performed immunohistochemistry on tissue microarrays made from a collection of 162 bladder tumor samples, composed mainly of HGT1 (102 cases) but also containing 41 low-grade tumors and 19 MIBC^53^. Tissue sections were deparaffinized in xylene and hydrated using an ethanol and water series. After antigen retrieval, the slides were treated with 3% H2O2 in PBS for 10 min to quench endogenous peroxidases, washed, and incubated in blocking solution (PBS containing 1% BSA and 1% Tween-20) for 1h at ambient temperature. Slides were incubated with TP63(FLEX Monoclonal Mouse Anti-Human p63 Protein, VENTANA anti-p63 (4A4), Part Number:GA66261-2) KRT5 (KRT 5 VENTANA anti-Cytokeratin 5/6 (D5/16B4) Mouse Monoclonal Primary Antibody), KRT20 (KRT 20 VENTANA anti-Cytokeratin 20 (SP33) Rabbit Monoclonal Primary Antibody) (Ab diluted in blocking solution for 1 h. Slides were washed in PBS and incubated with the peroxidase-based EnVision Kit (Dako).

### Computational and statistical analysis

#### H3K27Ac FiTAc-seq analysis

Alignment and peak calling: All samples were processed through the computational pipeline developed at the Dana-Farber Cancer Institute Center for Functional Cancer Epigenetics using open-source programs (https://github.com/liulab-dfci/CHIPS) 27. Sequence reads were aligned with the Burrows-Wheeler Aligner (BWA) 65 to build hg19, and uniquely mapped, nonredundant reads were retained. These reads were used to generate binding sites with ModelBased Analysis of ChIP-Seq 2 (MACS v2.1.1.20160309), with a q-value (FDR) threshold of 0.01 66. We evaluated multiple quality control criteria based on alignment information and peak quality: (i) sequence quality score; (ii) uniquely mappable reads (reads that can only map to one location in the genome); (iii) uniquely mappable locations (locations that can only be mapped by at least one read); (iv) peak overlap with Velcro regions, a comprehensive set of locations – also called consensus signal artifact regions – in the genome that have anomalous, unstructured high signal or read counts in next-generation sequencing experiments independent of cell line and of type of experiment; (v) number of total peaks (the minimum required was 8,000); (vi) high confidence peaks (the number of peaks that are tenfold enriched over background); (vii) Overlap with known DHS sites derived from the ENCODE Project (the minimum required was an 80% overlap); and (viii) peak conservation (a measure of sequence similarity across species based on the hypothesis that conserved sequences are more likely to be functional). Genome tracks were visualized using IGV (v2.14.1) 67.

Unsupervised analysis of the H3K27ac dataset: We used Permutational Multivariate Analysis of Variance (PERMANOVA) to statistically assess differences among the three clusters identified through hierarchical clustering of gene expression data. The PERMANOVA is a non-parametric method that evaluates whether the centroids of predefined groups differ significantly in multivariate space. This analysis was conducted using the ‘Adonis2’ function from the ‘vegan’ package in R, which operates on distance matrices and employs permutation tests to determine the statistical significance. By applying PERMANOVA, we tested the null hypothesis that there are no differences in the multivariate centroids among the three clusters. The analysis was performed with 999 permutations to assess the significance of the observed differences. This approach provided a robust statistical framework to validate the clustering results and ensure that the observed groupings reflected meaningful biological variation rather than random chance.

Differential binding analyses: The COBRA pipeline was used for differential analysis 27. Briefly, peaks from all samples were merged to create a union set of sites for each transcription factor and histone mark using Bedops 68. Read densities were calculated for each peak for each sample and used to compare cistromes across samples. Sample similarity was determined by hierarchical clustering using Spearman correlation between samples with significant differences. PCA plots were generated using standard R tools. Differential peaks were identified by DEseq2 with adjusted P ≤ 0.05 and |log2FoldChange| > 0.5. The total number of reads in each sample was applied to the size factor in DEseq2, which normalizes the sequencing depth between samples. Peaks from each group were used for motif analysis using the motif search findMotifsGenome.pl in HOMER2 (v3.0.0) 28 with a cutoff q-value ≤ 1e-10. The signals of each sample on the different binding sites were visualized using Deeptools 28,69. For the PCA of H3K27Ac signals in Figure 1B, all peaks from H3K27Ac ChIP-seq data were used.

Cistrome toolkit and GREAT analysis: We utilized the 'cistrome toolkit' web tool 70,71 to predict which factors exhibit significant binding overlap with differential peaks between URO1 (LLI) and URO2 (BL). Differential peaks were also analyzed using the GREAT web tool 33 to predict functions. A threshold for the single nearest genes within 400 kb was set. The prediction results for the GO biological processes were visualized using ggplot2 72.

#### Bulk RNA-seq analysis

Quality control and differential analysis: Read alignment, quality control, and data analysis were performed using the Visualization Pipeline for RNA-seq (VIPER) 73. Alignment to the hg19 human genome was done using STAR v2.7.0f 74 followed by transcript assembly using cufflinks v2.2.1 74,75 and RseQC v2.6.2 76. Differential gene expression analyses were performed by comparing LLI with BL using DESeq2 v1.18.1 76,77 utilizing absolute gene counts for RNA-Seq data and raw read counts for transcriptomic profiling data. The samples in the LLI and BL groups were matched with samples from H3K27ac ChIP-seq analysis. Specifically, in the H3K27ac dataset, there were six samples assigned to URO1 (LLI) and six to URO2 (BL), while in the RNA-seq cohort, there were three samples for URO1 (LLI) and four for URO2 (BL).

#### Clinical analysis

CDS development: To develop a robust classifier reflecting the epigenetic states of HGT1 tumors, we integrated H3K27ac FitAc-seq data with matched bulk RNA-seq profiles. The derivation process followed a systematic multi-step pipeline. First, we identified subtype-specific regulatory elements by performing differential enrichment analysis of H3K27ac signals between the URO1, URO2, and MP clusters. High-confidence peaks were defined using a q-value (FDR) threshold of less than 0.01. These differentially enriched H3K27ac regions were then linked to their predicted target genes and cross-referenced with differential expression data from matched RNA-seq samples. Candidate genes for the BL and LLI signatures were selected based on a differential expression magnitude of |log2FC| > 5, while genes for the MP signature were selected using a threshold of |log2FC| > 2, as the MP subtype exhibited more nuanced transcriptional differences compared to the highly divergent BL and LLI states. All candidate genes were required to have an adjusted P-value < 0.05 and a direct association with a subtype-specific H3K27ac-enriched region. To determine the optimal number of features for the Chromatin-Derived Score (CDS) classifier, we performed iterative testing of signature sizes ranging from 5 to 25 genes. We found that selecting 15 genes per subtype provided the highest classification accuracy while maintaining statistical parity across all three states.

HGT1 and UROMOL bulk RNA-seq scoring and statistics: The Gene set variation analysis (GSVA) 79 score was computed for both HGT1 and UROMOL RNA-seq cohorts, and the differences between the GSVA scores of LLI and BL were used for sample classification. Samples falling within the 1st quartile, reflecting the highest LLI to BL score, were classified as LLI-like, while those within the 4th quartile were categorized as BL. Boxplots representing marker expression in the BL and LLI groups were plotted using the ggplot2 v3.5.1. GSVA was also conducted using data from the UROMOL cohort. Samples with LLI–BL > 0 were classified as LLI-like, while those with LLI–BL < 0 were designated as BL-like. For the UROMOL cohort, chi-square tests were conducted to assess the associations between the four classes defined in the UROMOL2021 classification and the two subtypes identified by our LLI–BL classification. Chi- square tests were also used to evaluate the association between tumor progression and tumor class.

Marker expression between LLI and BL cases was compared using Student’s t-test with Welch correction for the UROMOL cohort (n > 50, unequal variances; F-test p-value < 0.05) and the Wilcoxon rank-sum test for the HGT1 cohort (n < 50, non-Gaussian distribution; Shapiro test p-value < 0.05), with results visualized by boxplots. All statistical analyses were performed using the *stats* package (v4.3.2) in RStudio (R v4.3.2).

Cox proportional hazards analyses were performed using the survival package (v3.7.0), and Kaplan–Meier curves were plotted using ggsurvfit (v1.1.0). The log-rank test was used to compare survival distributions between groups.

Gene Set Enrichment Analysis (GSEA) was conducted using GSEA software (GSEA Java; v4.1.0) with Hallmark gene sets. Genes were pre-ranked based on log2 fold change (log2FC) for the BL versus LLI comparison, and enrichment scores were computed (p = 1, weighted). The top 10 enriched gene sets on each side were visualized using ggplot2 (v3.5.1) 78.

#### IHC staining scoring

The scoring of the immunohistochemical analysis of the collection of 162 bladder tumor samples included in TMAs was performed by an expert pathologist. p63 staining was assessed using Histoscore (H-score), a metric that considers both the percentage of stained cells and staining intensity. The H-score was calculated as follows: 3 × percentage of strongly stained nuclei + 2 × percentage of moderately stained nuclei + percentage of weakly staining nuclei. Intensity was scored as follows: 0, no staining; 1, weak staining; 2, moderate staining; and 3, strong staining. For KRT 5/6 and KRT20, the percentage of positive cells was assessed.

#### Single-cell ATAC-seq data processing

Quality control: The scATAC-seq data from two samples (URO1 - 7166 cells; URO2 - 1733 cells) were processed using the cellranger-atac (v2.0.0) 80 pipeline with default parameters. Quality control filtering of low-quality cells was performed using the R packages Seurat (v3) 81 and Signac (v1.6.0) 81,82 based on criteria including nucleosome-binding pattern strength, transcription start site enrichment score, number of fragments in peaks>100, and peaks > 600.

Sample integration: Integration of samples was achieved using a common peak set derived from the peaks of each sample with peak widths ranging from 20 to 10,000. The peak count matrix was subjected to TF-IDF normalization using the Signac package. Singular value decomposition (SVD), also referred to as latent semantic indexing (LSI), was performed on the normalized matrix using the RunSVD function in Signac, resulting in 2:30 LSI components. These components were then utilized for nonlinear dimensionality reduction using the RunUMAP function from the Seurat package. The Seurat function FindNeighbors was used to create a shared nearest neighbor graph based on the 2:30 LSI components. Subsequently, the FindClusters function was employed to iteratively cluster nuclei and optimize modularity using the Louvain algorithm. The gene activity score for the integrated object was computed using the GeneActivity function, and subsequently normalized using the NormalizeData function. Differential analysis for each cluster was conducted using the FindMarkers function, whereas differential motifs for each cluster were identified using chromVar. The LLI and BL signature scores were computed using the AddModuleScore function. Motif enrichment analysis for each cluster was performed using ChromVAR 83.

Single-cell ATAC cell type annotation: The scATAnno 45 was used to perform cell type annotation.

Single-cell ATAC CNV inference: Our internal CNV calling tool was used to call copy number variation from single-cell ATAC-seq samples, which adapted an existing bulk ATAC-seq method to utilize off-target scATAC-seq reads to infer DNA copy number changes. The genome was divided into large intervals and the coverage of each interval was determined. The scATACCNV averaged the coverage of 100 GC-matched intervals to establish background levels. Comparisons between interval coverage and the corresponding GC-matched background enabled the estimation of CNV fold changes. Interval size of 1 Mb was chosen to accommodate the sparse scATAC-seq data using the "ChunkGRanges" function in Genomics Range. The "GCcontent" function of biovizBase was used to calculate GC content for each interval. The coverage adjustment for the removed peaks was achieved by incorporating the effective window size during the calculation.

#### Fixed snRNA-seq (FLEX) data processing

Quality control, dimensionality reduction and clustering: Cellranger (version 6.0.2) 84 using 10x Genomics aligned reads to the prebuilt GRCh38 genome reference version 2020-A (refdatagex-GRCh38-2020-A). The R package Seurat was used to perform subsequent processing using the cell-by-gene matrix from the cellranger. Doublets were identified and removed using the scDblFinder 85 package. The barcodes were filtered using UMIs, expressed genes, and mitochondrial gene percentages. Cell cycle and mitochondrial genes were regressed out using ScaleData function. The filtered gene-count matrix underwent scaling and normalization for sequencing depth using Seurat’s ‘SCTransform' function. The principal components were then calculated using Seurat's RunPCA function. The cells were then clustered using a Louvain graph-based approach. Initially, the Seurat function FindNeighbors was employed to construct a k-nearest neighbor graph based on Euclidean distances in principal component analysis (PCA) space. Cells with similar expression patterns are connected by edges. The first 30 principal components were used in this step with default parameters. Clustering was then performed using modularity optimization techniques, specifically the Louvain algorithm from the Seurat FindClusters function.

Data integration and snRNA-seq cell type annotation: After quality control, the nine filtered samples were merged. Subsequently, the merged objects were normalized using the Seurat SCTransform function, employing the same parameters as when normalizing the individual objects. Cells were then clustered using the top 30 PCA dimensions via the FindNeighbors and FindClusters functions, with the resolution parameter set to 0.2. Next, the RunUMAP function is applied to obtain new cell embeddings. Cell type annotation was performed on nine transcriptionally distinct clusters using Seurat-derived marker genes and SingleR. For each cluster, we identified differentially expressed genes with FindAllMarkers and assigned identities based on canonical markers per lineage: tumor/epithelial cells (*KRT7*, *KRT19*, *FGFR3*, *GATA3*), fibroblasts (*COL1A1*, *COL1A2*, *COL3A1*, *DCN*), macrophages/monocytes (*CD163*, *CSF1R*, *MS4A6A*, *AEBP1*), endothelial cells (*PECAM1*, *VCAM1*, *VASH1*, *EGFL7*), B cells (*MS4A1*, *CD37*, *BANK1*, *MZB1*), T cells (*CD3D*, *CD3E*, *CD4*, *IL7R*), and plasma cells (*MZB1*, *XBP1*, *SDC1*, *PRDM1*). In parallel, SingleR was run on the normalized expression matrix using appropriate reference datasets to obtain automated cell type labels at the single-cell level. Cluster-level annotations were finalized by integrating SingleR 83 predictions with the marker-based assignments, and only labels that were concordant between the two approaches and biologically plausible in the disease and tissue context were retained; clusters with discordant or ambiguous profiles were left unannotated or grouped as other. Finally, for epithelial clusters, we also integrated copy number profiles obtained from inferCNV to support the distinction between malignant tumor cells and non-malignant epithelial populations. The LLI and BL signature scores were calculated using the AddModuleScore function. InferCNV (https://github.com/broadinstitute/inferCNV)(v1.19.1) was used to identify CNA in single cells with threshold: cutoff = 0.1, HMM = TRUE, leiden_method="simple,” cluster_by_groups=TRUE, denoise=TRUE.

#### Classification, GSEA

Tumor cells were classified based on their signature scores. Cells were ranked using LLI and BL scores, and a quantile threshold was established. Cells in the 1st quantile of LLI that did not meet the BL threshold were classified as LLI-like. Similarly, cells meeting the BL, but not the LLI threshold, were classified as BL-like. The remaining cells were labeled as unidentified. The ssGSEA analysis was performed using ‘escape’ in scRepertoire toolkit for 3 classes with hallmark gene sets 86.

#### CAF subtype classification and macrophage signature scoring

CAF subtype classification and macrophage signature scoring of snRNA-seq cancer-associated fibroblast subtypes were classified by expression of *FN1* (myCAFs), *C3* (iCAFs), *CD74* (apCAFs), *SLC14A1* (IFN CAF) and *PSCA* (PSCA+ CAF). Macrophage polarization was assessed by calculating M1 and M2 signature scores using the Add ModuleScore function in Seurat, based on established gene sets: M1 marker - *CD80*, *CD86*, *NOS2*, *IL12B*, *IL1B*, *CXCL9*, *CXCL10*, *CXCL11*,* EMP1* 87,88; M2 marker - *CD163*,* ARG1*, *IL10*, *TGFB1*, *C1QA*, *C1QB*, *C1QC*, *MMP9* 87,89.

#### IHC image analysis

Alignment: Staining for KRT5 and KRT20 was performed on two adjacent slides of the same FFPE block for each tumor. The StackReg plug-in (https://bigwww.epfl.ch/thevenaz/stackreg/) was used in FiJi (v1.53t) to align the images. The same filters as previously described were used to detect KRT5 and KRT20 positive regions. Image annotation was performed using RStudio (R v4.3.2).

Distances: Nineteen images of tumors positive for both KRT5 and KRT20 (36% of the mixed tumors) were analyzed to measure the distance between KRT20-positive or KRT5-positive) cells and the vascular stroma. Briefly, for each image, the vascular stroma was outlined manually (under the supervision of) a patho-histologist using FiJi (v1.53t). Each colored image was then converted to black and white, and stained areas were detected using the “Set Auto Threshold'' tool with the “Moments''algorithm. Because nuclei were stained with hematoxylin, they appeared dark blue and were thus detected as part of the KRT5 or KRT20 positive areas. To distinguish the diaminobenzidine signal (marking the KRT20 or KRT5 cells) from the hematoxylin signal (marking nuclei), an additional filter was applied to remove pixels that were more blue than brown from the KRT5+/KRT20+ region. This was performed in RStudio by removing pixels with a higher blue intensity than the red one. Finally, for each pixel in the stained area, the closest blood vessel pixel was identified using Python’s R-tree library, and the Euclidean distance between them was measured. Image annotations and paired dot plots were performed using RStudio (R v4.3.2). The Wilcoxon rank-sum test was performed to compare distances between KRT5/KRT20 positive pixels and the vascular stroma.

#### Spatial transcriptomics data processing and analysis

Raw sequencing reads were processed using the 10x Genomics Space Ranger HD pipeline (version 3.1.3), which included alignment to the GRCh38 human reference genome and assignment of reads to spatial barcodes. Gene expression matrices were generated at 2-µm resolution. Cell segmentation was performed with bin2cell 90 using the binned output from Space Ranger as the input, with parameters: mpp = 0.5, prob_thresh=0.1, nms_thresh=0.5.

Segmented cell-level gene expression data were analyzed using Scanpy (version 1.10.2) 54. Low-quality cells and lowly expressed genes were filtered based on gene count, UMI count, and mitochondrial RNA content. The data were normalized, log-transformed, and scaled. Highly variable genes were selected using Seurat v3. Principal component analysis was performed, followed by neighborhood graph construction, UMAP dimensionality reduction, and Leiden clustering to identify transcriptionally distinct cell populations.

#### Cell type annotation for spatial transcriptomics

Cell types were assigned by integrating automated reference-based predictions from CellTypist 91 with manual curation based on differentially expressed marker genes from Leiden clusters. Marker genes were identified via differential expression analysis and cell identities were confirmed using known lineage markers and tissue context.

#### CAF subtype classification and macrophage signature scoring of spatial transcriptomics

CAF subtype classification and macrophage signature scoring in the spatial transcriptomics data were performed using the same CAF marker genes and M1/M2 macrophage gene signatures as defined for the scRNA-seq analysis (see “CAF subtype classification and macrophage signature scoring” above). Spatial co-occurrence between CAFs and other cell types was quantified using the `gr.co_occurrence()` function in Squidpy, and macrophage polarization was assessed by calculating M1 and M2 signature scores in spatial spots using the `tl.score_genes()` function in Scanpy, applying the same gene signatures and scoring procedure as in the single-cell dataset.

#### Spatial transcriptomics visualization

Data visualization was performed using Scanpy and Squidpy 54,55 plotting functions, including UMAP embeddings, spatial feature plots, and co-occurrence curves to illustrate cellular distribution and spatial interactions.

## Supplementary Material

Supplementary figures and tables.
