# Supplementary material for: Epigenetic subtypes of high-grade T1 bladder cancer reveal intra-tumor heterogeneity and distinct interactions with tumor microenvironment
Source: Theranostics. 2026 May 18;16(12):6892–910. doi: 10.7150/thno.129729 (PMC13232474; doi:10.7150/thno.129729)

Table S1-metadata

samples	scATAC	visiumHD	scRNA-seq	K27ac	RNA-seq	stage	material	histology
HGT1_1	yes						FF	URO
HGT1_2	yes						FF	URO
mpbc1		yes				MIBC	FFPE	URO+MP
mpbc2		yes				MIBC	FFPE	URO+MP
vh176			yes	yes	yes	NMIBC-HGT:	FFPE	URO
vh61			yes	yes	yes	NMIBC-HGT:	FFPE	URO
vh12			yes		yes	NMIBC-HGT:	FFPE	URO
vh122			yes		yes	NMIBC-HGT:	FFPE	URO
vh125			yes		yes	NMIBC-HGT:	FFPE	URO
vh15			yes		yes	NMIBC-HGT:	FFPE	URO
vh24			yes		yes	NMIBC-HGT:	FFPE	URO
vh1			yes	yes		NMIBC-HGT:	FFPE	URO
vh75			yes			NMIBC-HGT:	FFPE	URO
vh100				yes	yes	NMIBC-HGT:	FFPE	URO
vh126				yes	yes	NMIBC-HGT:	FFPE	URO
vh21				yes	yes	NMIBC-HGT:	FFPE	URO
vh81				yes	yes	NMIBC-HGT:	FFPE	URO
vh93				yes	yes	NMIBC-HGT:	FFPE	URO
vh96				yes	yes	NMIBC-HGT:	FFPE	URO
vh101					yes	NMIBC-HGT:	FFPE	URO
vh102					yes	NMIBC-HGT:	FFPE	URO
vh103					yes	NMIBC-HGT:	FFPE	URO
vh105					yes	NMIBC-HGT:	FFPE	URO
vh112					yes	NMIBC-HGT:	FFPE	URO
vh113					yes	NMIBC-HGT:	FFPE	URO
vh116					yes	NMIBC-HGT:	FFPE	URO
vh121					yes	NMIBC-HGT:	FFPE	URO
vh123					yes	NMIBC-HGT:	FFPE	URO
vh134					yes	NMIBC-HGT:	FFPE	URO
vh135					yes	NMIBC-HGT:	FFPE	URO
vh14					yes	NMIBC-HGT:	FFPE	URO
vh140					yes	NMIBC-HGT:	FFPE	URO
vh144					yes	NMIBC-HGT:	FFPE	URO
vh145					yes	NMIBC-HGT:	FFPE	URO
vh146					yes	NMIBC-HGT:	FFPE	URO
vh150					yes	NMIBC-HGT:	FFPE	URO
vh151					yes	NMIBC-HGT:	FFPE	URO
vh159					yes	NMIBC-HGT:	FFPE	URO
vh16					yes	NMIBC-HGT:	FFPE	URO
vh163					yes	NMIBC-HGT:	FFPE	URO
vh165					yes	NMIBC-HGT:	FFPE	URO
vh171					yes	NMIBC-HGT:	FFPE	URO
vh180					yes	NMIBC-HGT:	FFPE	URO
vh184.c					yes	NMIBC-HGT:	FFPE	URO
vh19					yes	NMIBC-HGT:	FFPE	URO
vh29					yes	NMIBC-HGT:	FFPE	URO
vh30					yes	NMIBC-HGT:	FFPE	URO
vh31					yes	NMIBC-HGT:	FFPE	URO
vh32					yes	NMIBC-HGT:	FFPE	URO
vh34					yes	NMIBC-HGT:	FFPE	URO
vh42					yes	NMIBC-HGT:	FFPE	URO
vh45					yes	NMIBC-HGT:	FFPE	URO
vh46					yes	NMIBC-HGT:	FFPE	URO
vh49					yes	NMIBC-HGT:	FFPE	URO
vh5					yes	NMIBC-HGT:	FFPE	URO
vh52					yes	NMIBC-HGT:	FFPE	URO
vh54					yes	NMIBC-HGT:	FFPE	URO
vh55					yes	NMIBC-HGT:	FFPE	URO
vh56					yes	NMIBC-HGT:	FFPE	URO
vh62					yes	NMIBC-HGT:	FFPE	URO
vh66					yes	NMIBC-HGT:	FFPE	URO
vh68					yes	NMIBC-HGT:	FFPE	URO
vh7					yes	NMIBC-HGT:	FFPE	URO
vh73					yes	NMIBC-HGT:	FFPE	URO
vh78					yes	NMIBC-HGT:	FFPE	URO
vh79					yes	NMIBC-HGT:	FFPE	URO
vh91					yes	NMIBC-HGT:	FFPE	URO
vh97					yes	NMIBC-HGT:	FFPE	URO
MP1				yes			FFPE	MP
MP3				yes			FFPE	MP
MP5				yes			FFPE	MP
MP6				yes			FFPE	MP
MP8				yes			FFPE	MP
MP9				yes			FFPE	MP
vh127				yes			FFPE	MP
vh77				yes			FFPE	MP

Table S2. FiTAc-seq information

Sample	UniqMappedReads(M)	TotalPeaks	FRiP	DHS_%
MP1	57.6	15503	4.4	85.34
MP3	47	40979	7.9	94.1
MP5	50.7	38726	10.7	96.7
MP6	64	56555	36.6	99.3
MP8	56.5	21048	6.4	96.12
MP9	96.2	52180	23.5	99.28
UCC1	36.3	41461	23.8	99.34
UCC10	154.9	29937	13.7	95.32
UCC11	74.4	31915	16	95.82
UCC12	75.5	29122	17.4	97.4
UCC13	152.5	40480	15.9	95.7
UCC3	73.2	10606	3.1	90.42
UCC4	78.3	39672	20.5	98.78
UCC5	80.2	44241	25.6	99.5
UCC7	79.8	53933	29.7	99.66
UCC8	57	53594	33.3	99.36
UCC9	89.7	33869	19.1	96.86

Table S3. Motif analysis

MP vs URO

MP						
Name	PValue	log(PValue)	# Target Sequ	% of Targets S	# Backgroun	% of Background Sequences with Motif
Jun-AP1(bZIP)/K562-cJun-ChIP-Seq(	1.00E-07	-17.703283	278	9.75%	3205	6.98%
GRHL2(CP2)/HBE-GRHL2-ChIP-Sec	1.00E-03	-8.608498	442	15.51%	6055.5	13.18%
Unknown3/Drosophila-Promoters/H	1.00E-02	-6.194808	92	3.23%	1082.9	2.36%
TFE3(bHLH)/MEF-TFE3-ChIP-Seq(G	1.00E-02	-5.241718	79	2.77%	940.1	2.05%
RFX(HTH)/K562-RFX3-ChIP-Seq(SR	1.00E-02	-6.567761	69	2.42%	757.2	1.65%

URO						
Name	PValue	log(PValue)	# Target Seq	% of Targets	# Backgroun	% of Background Sequences with Motif
p73(p53)/p6	1.00E-173	-399.82086	675	11.15%	1373.7	3.11%
IRF2(IRF)/Er	1.00E-26	-60.973941	700	11.56%	3372.9	7.64%
Ets1-distal(E	1.00E-22	-51.614331	1529	25.25%	8848.5	20.03%
Bach1(bZIP)/	1.00E-16	-38.368215	346	5.71%	1564.1	3.54%
Tcfcp2l1(CP2	1.00E-15	-35.744305	743	12.27%	4033	9.13%

MP + URO-LLI						
Name	PValue	log(PValue)	# Target Sequ	% of Targets S	# Backgroun	% of Background Sequences with Motif
Hnf1(Homeobox)/Liver-Foxa2-Chip-	1.00E-07	-16.192816	261	11.36%	3818	8.21%
GRHL2(CP2)/HBE-GRHL2-ChIP-Sec	1.00E-04	-10.903541	497	21.64%	8477.4	18.22%
FAR1(FAR1)/col-FAR1-DAP-Seq(GSI	1.00E-03	-7.455878	51	2.22%	629.9	1.35%
GT1(Trihelix)/col-GT1-DAP-Seq(GSE	1.00E-03	-8.266723	13	0.57%	8.6	0.19%
GATA3(Zf),DR8/iTreg-Gata3-ChIP-S	1.00E-03	-7.484742	106	4.61%	153.5	3.32%

URO-BL						
Name	PValue	log(PValue)	# Target Seq	% of Targets	# Backgroun	% of Background Sequences with Motif
p73(p53)/p6	1.00E-211	-487.82993	901	10.24%	1265.9	3.05%
Jun-AP1(bZIF	1.00E-141	-326.93713	2290	26.03%	6429	15.48%
Tcfcp2l1(CP2	1.00E-32	-75.316065	1052	11.96%	3417.7	8.23%
GFY-Staf(?,Z	1.00E-13	-31.923202	309	3.51%	920.8	2.22%
RORg(NR)/L	1.00E-13	-30.50067	839	9.54%	3065.9	7.38%

MP + URO-BL						
Name	PValue	log(PValue)	# Target Seq	% of Targets	# Backgroun	% of Background Sequences with Motif
Jun-AP1(bZIP)/K562-cJun-ChIP-Seq(	1.00E-71	-164.14494	564	39.14%	9219.6	18.80%
p73(p53)/p63/Trachea-p73-ChIP-Se	1.00E-18	-43.563735	128	8.88%	1786	3.64%
Ets1-distal(ETS)/CD4+-PolII-ChIP-Se	1.00E-07	-17.527748	436	30.26%	11736.6	23.93%
Tcfcp2l1(CP2)/mES-Tcfcp2l1-ChIP-S	1.00E-05	-12.255455	197	13.67%	4893.1	9.98%
RAR:RXR(NR),DR5/ES-RAR-ChIP-Se	1.00E-04	-10.040651	56	3.89%	1074.5	2.19%

URO-LLI						
Name	PValue	log(PValue)	# Target Seq	% of Targets	# Backgroun	% of Background Sequences with Motif
IRF2(IRF)/Er	1.00E-18	-43.435107	202	16.37%	4112.1	8.42%
Ets1-distal(E	1.00E-16	-37.704382	376	30.47%	9958.7	20.39%
ETS:RUNX(E	1.00E-07	-16.576669	143	11.59%	3582.7	7.34%
T1ISRE(IRF)/	1.00E-04	-10.597738	27	2.19%	431.4	0.88%
GATA:SCL(Zf	1.00E-04	-10.48091	156	12.64%	4462.6	9.14%

URO-BL						
Name	PValue	log(PValue)	# Target Sequ	% of Targets S	# Backgroun	% of Background Sequences with Motif
Jun-AP1(bZIP)/K562-cJun-ChIP-Seq(	1.00E-241	-555.97164	2673	28.20%	6073.2	14.87%
p73(p53)/p63/Trachea-p73-ChIP-Se	1.00E-161	-371.82482	832	8.78%	1213.3	2.97%
Tcfcp2l1(CP2)/mES-Tcfcp2l1-ChIP-S	1.00E-27	-63.065982	1067	11.26%	3281.9	8.03%
ETS(ETS)/Promoter/Homer	1.00E-18	-43.739891	1818	19.18%	6418	15.71%
RAR:RXR(NR),DR5/ES-RAR-ChIP-Se	1.00E-08	-18.982154	256	2.70%	757.3	1.85%

URO-LLI						
Name	PValue	log(PValue)	# Target Seq	% of Targets	# Backgroun	% of Background Sequences with Motif
Hnf1(Homeo	1.00E-10	-23.251818	425	11.95%	4068.5	8.76%
IRF2(IRF)/Er	1.00E-09	-22.953174	295	8.30%	2631.1	5.66%
GRHL2(CP2)/	1.00E-08	-19.147771	857	24.10%	9356.9	20.14%
GATA(Zf),IR4	1.00E-05	-12.969869	210	5.91%	1978.9	4.26%
Ets1-distal(E	1.00E-04	-9.461224	574	16.14%	6452.7	13.89%

Table S4. LLI and BL signatures

LLI	BL
FBN2	BMP7
SLC4A4	ERN2
CTTNBP2	SRPX2
KALRN	UGT1A10
SELL	CLCA2
PMP22	SPOCD1
UGT2B7	SERPINB5
COL12A1	SEMA4B
PTH2R	CLCA4
ANKRD36	CLU
DCDC2	UGT1A7
PRTG	SH3PXD2A
BPGM	AQP3
PDE9A	ANXA10
ANXA3	SLITRK6

Table S5. MP signature

**MP**
MUC4
ANPEP
MAL
MMP7
COL4A4
PLCXD3
SPNS2
AREG
FAT3
ASS1
FBN2
BPGM
TMC5
LIPC
ELOVL7

Supplementary Figure 1

A) Heatmap representation of the differential H3K27ac regions distinguishing MP, URO2 and URO1 clusters. B) Results of motif enrichment analysis performed on MP-specific differential peaks. C) Signal distribution of H3K27ac marked enhancers in representative URO1 and URO2 samples. Representative super-enhancers identified using the ROSE algorithm are indicated. D) Integrated UMAP visualization of the two scATAC-seq datasets (HGT1_1 and HGT1_2), colored by sample origin. E) Copy-number variation (CNV) analysis of tumor cells from the two scATAC-seq samples (HGT1_1 and HGT1_2). Rows represent individual cells clustered using K-means. Green bars indicate normal cells, whereas purple bars indicate tumor cells. The color scale represents relative CNV signal centered around neutral copy number. Red indicates copy-number gains and blue indicates copy-number losses. F) Annotation of normal cell populations identified in the scATAC-seq datasets, as described in Methods. G) Integrated UMAP visualization of HGT1_1 and HGT1_2 showing enrichment of the basal-like (BL, red, top) and luminal-like inflammatory (LLI, blue, bottom) chromatin signature scores. Color scales indicate relative enrichment values. H) Integrated UMAP visualization showing accessibility at the KRT5 and KRT20 promoter regions. I) Integrated UMAP visualization showing enrichment of TP63 and GATA3 transcription factor motifs. J) Integrated UMAP analysis of HGT1_1 and HGT1_2 showing transcription factor motif enrichment across clusters defined using the Louvain graph-based clustering algorithm, as described in Methods.

Supplementary Figure 2

A) Boxplots showing expression levels of representative marker genes in tumors classified as basal-like (BL, red) or luminal-like inflammatory (LLI, blue) in the HGT1 cohort. Groups were defined using the upper and lower quartiles of the corresponding subtype signature scores. P-values were calculated using two-sided t-tests. B) Boxplots showing expression levels of representative marker genes in tumors classified as basal-like (BL, red) or luminal-like inflammatory (LLI, blue) in the UROMOL cohort. Groups were defined using the upper and lower quartiles of the corresponding subtype signature scores. P-values were calculated using two-sided t-tests.

Supplementary Figure 3

A) UMAP visualization of all eleven snRNA-seq datasets comprising 37,879 nuclei, colored by sample ID. B) UMAP visualization showing log-normalized NECTIN4 expression levels (color scale). C) CNV analysis of two representative tumors analyzed by snRNA-seq (vh75, left; vh125, right). The color scale represents relative copy-number variation (CNV) signal centered around neutral copy number. Red indicates copy-number gains, whereas blue indicates copy-number losses. D) UMAP visualization of all eleven snRNA-seq datasets annotated by tumor and normal cell populations, including classification of normal cell types. E) Proportional bar plot showing the relative abundance of each cell type across tumor samples. F) Association between differential H3K27ac regions and differential gene expression in MP samples. The volcano plot depicts RNA-seq log2 fold-change (x-axis) versus adjusted p-value calculated by DESeq2 (y-axis). Each dot represents one gene. Yellow dots indicate significantly differentially expressed genes associated with nearby differential H3K27ac regions in MP samples, whereas gray dots indicate differentially expressed genes without nearby significant differential H3K27ac regions. G) UMAP visualization of all eleven snRNA-seq datasets showing log-normalized expression of KRT20 (left), KRT5 (middle), and TP63 (right) (color scales). H) UMAP visualization of cancer

cells from nine HGT1 tumors annotated according to the UROMOL2021 classification system. Cells were assigned to one of four UROMOL2021 classes: class 1 (orange), class 2a (light blue), class 2b (dark blue), and class 3 (red), while unclassified cells are shown in gray. No cells classified as class 2b were detected at the single-cell level. I) Summary of immunohistochemistry (IHC) scoring for KRT5 and KRT20 staining in the tissue microarray (TMA). Tumors were classified as KRT5-positive, KRT20-positive, KRT5/KRT20 double-positive, or double-negative (DN). J) Representative KRT5 and KRT20 staining demonstrating their distinct spatial distributions. Expanded views illustrate the anticorrelation between KRT5 and KRT20 staining and show the relative proximity of KRT5-positive cells (red arrows) and KRT20-positive cells (blue arrows) to blood vessels. K) Comparison of the mean distance to the nearest blood vessel for KRT5-positive and KRT20-positive cells in tumors positive for both markers (N = 16). KRT5-positive cells were significantly closer to blood vessels. L) Representative staining for KRT5, KRT20, and TP63 showing that TP63-high cells exhibit greater overlap with KRT5 expression than with KRT20.

Supplementary Figure 4

A) H&E staining of the two micropapillary bladder cancer (MPBC) cases analyzed by spatial transcriptomics. B) UMAP visualization showing cell type clustering for MPBC1 (left) and MPBC2 (right), as described in Methods. C) Spatial representation of CDS subtype scoring in MPBC1, showing enrichment of basal-like (BL, left), micropapillary (MP, middle), and luminal-like inflammatory (LLI, right) transcriptional programs. Color scales indicate relative enrichment values. D) Spatial representation of CDS subtype scoring in MPBC2, showing enrichment of basal-like (BL, left), micropapillary (MP, middle), and luminal-like inflammatory (LLI, right) transcriptional programs. Color scales indicate relative enrichment values. E) Spatial mapping of cancer-associated fibroblasts (CAFs) in MPBC1 reveals distinct stromal organization between the MP and URO components. The MP region (dashed area, left) contains a high density of CAFs interspersed with tumor cells, whereas the URO region (dashed area, right) shows minimal CAF infiltration. F) Spatial representation of canonical CAF marker expression in the MP and URO regions. These analyses demonstrate reduced CAF-associated programs in the URO component relative to the MP component. G) Top: UMAP visualization showing log-normalized EGLN3 expression across cancer cells from the eleven snRNA-seq datasets. Color scales indicate relative enrichment values. Bottom: violin plot showing EGLN3 expression in snRNA-seq cells classified as BL, LLI, or MP. EGLN3 expression is significantly enriched in BL cells relative to the other groups ($p < 0.001$, two-sided t-test).

Supplementary Figure 1

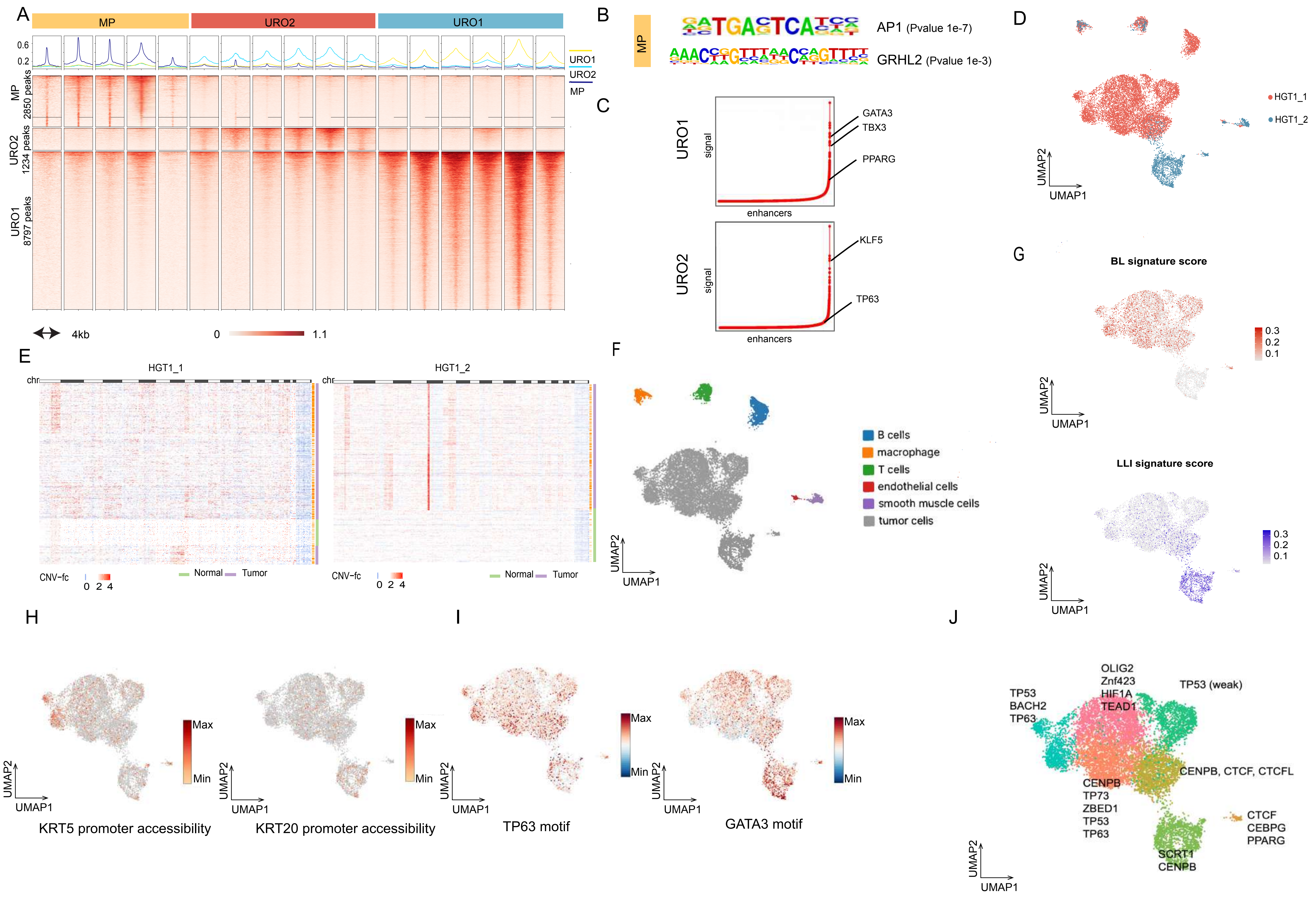

Supplementary Figure 2

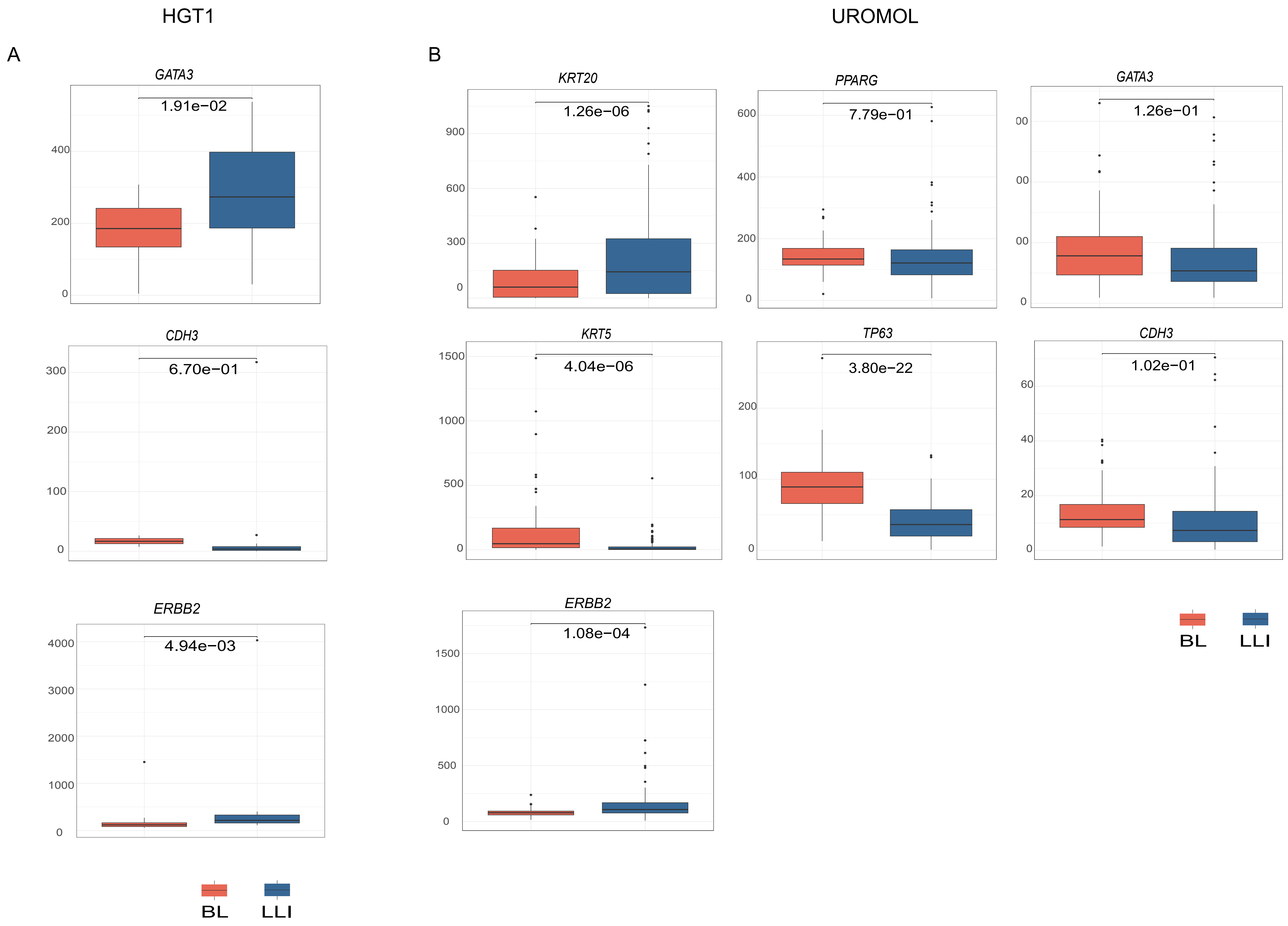

Supplementary Figure 3

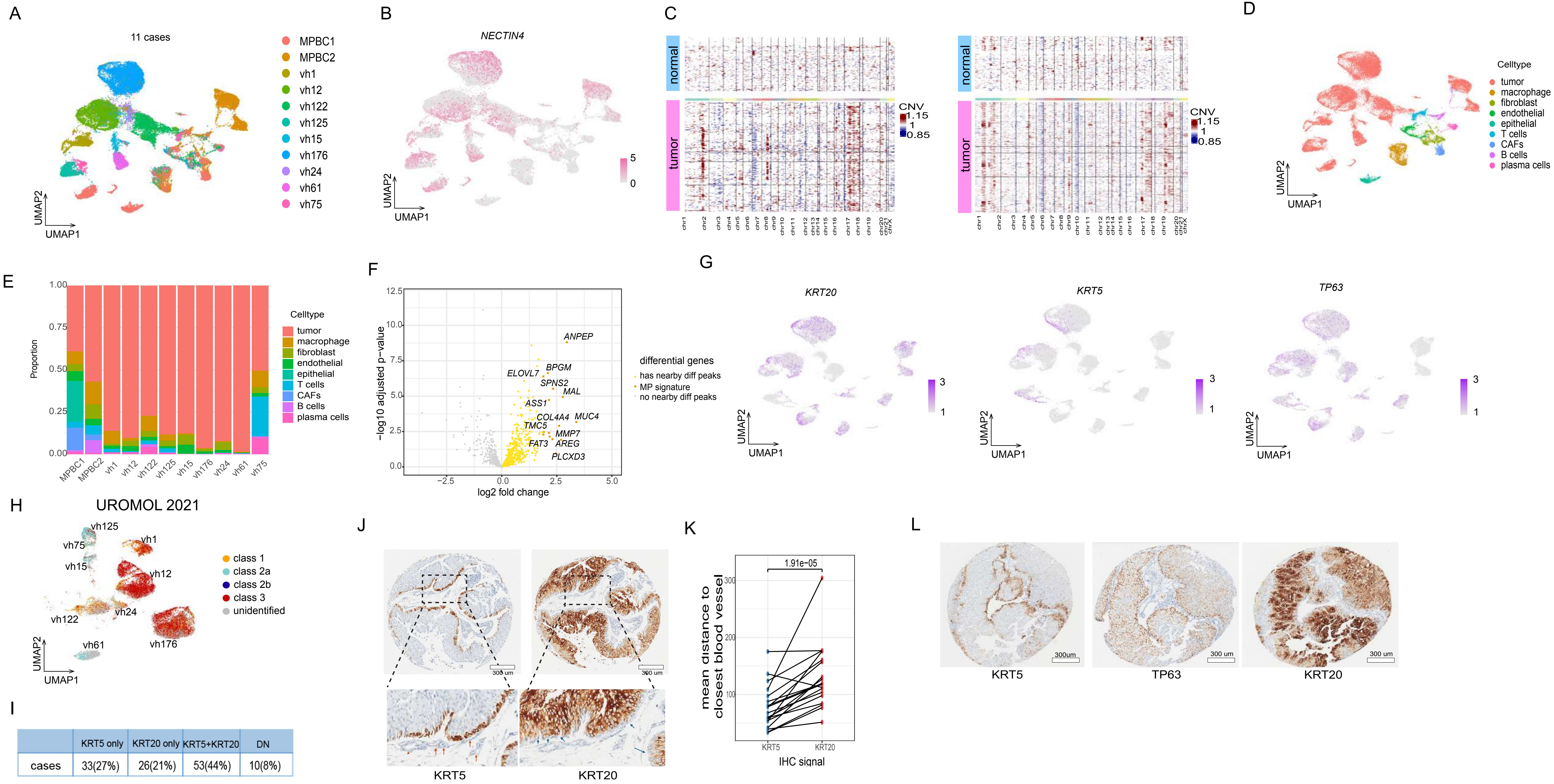

	KRT5 only	KRT20 only	KRT5+KRT20	DN
cases	33(27%)	26(21%)	53(44%)	10(8%)

Supplementary Figure 4

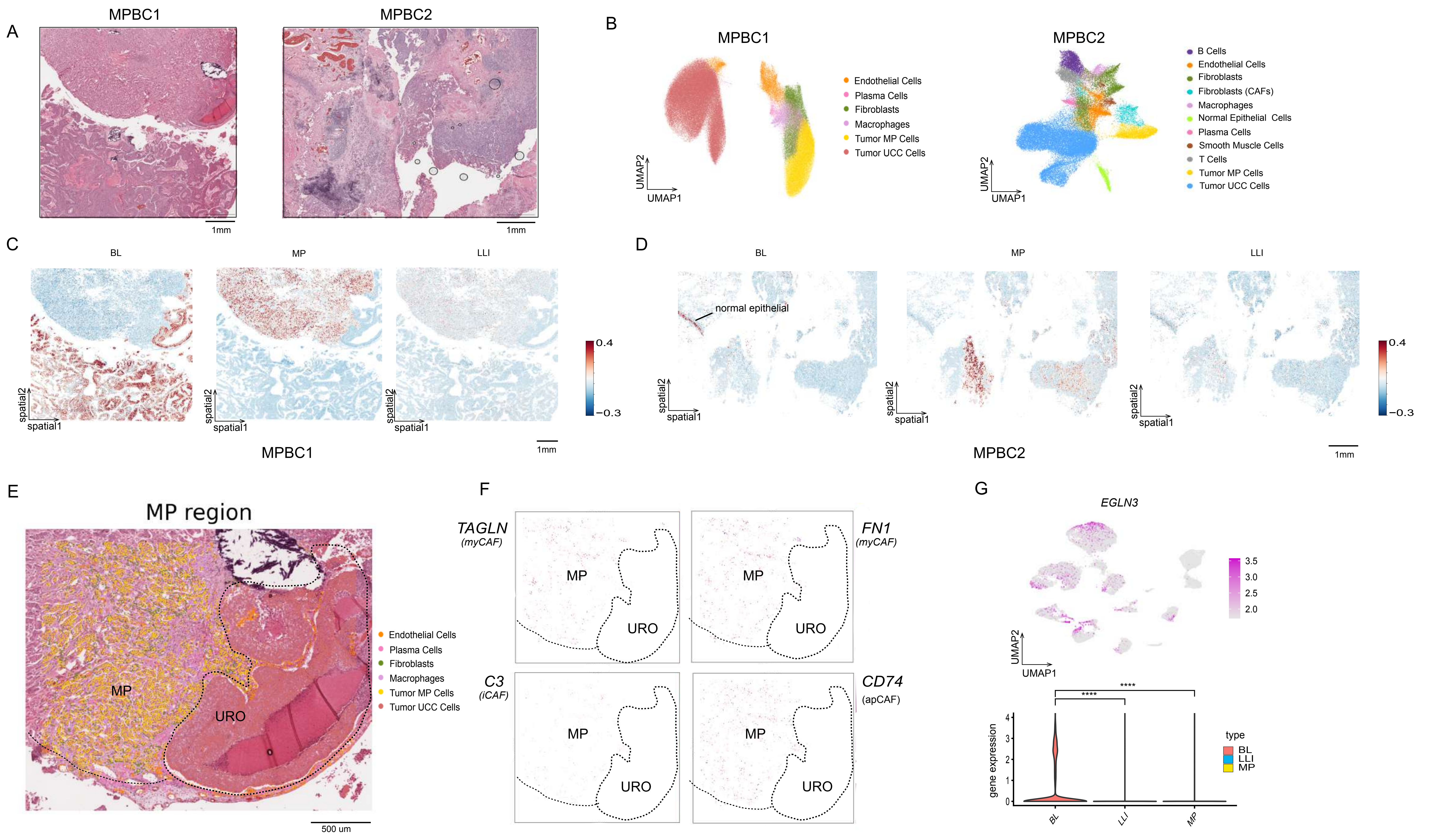

Supplement: Supplementary file 1 — Supplementary figures and tables. [file thnov16p6892s1.pdf]
